# Beyond VEGF: Angiopoietin–Tie Signaling Pathway in Diabetic Retinopathy

**DOI:** 10.3390/jcm13102778

**Published:** 2024-05-09

**Authors:** Genesis Chen-Li, Rebeca Martinez-Archer, Andres Coghi, José A. Roca, Francisco J. Rodriguez, Luis Acaba-Berrocal, María H. Berrocal, Lihteh Wu

**Affiliations:** 1 Asociados de Mácula Vitreo y Retina de Costa Rica, San José 60612, Costa Ricarmartinezar@outlook.com (R.M.-A.); soycoghi@hotmail.com (A.C.); 2 Oftalmologos Contreras, Lima 15036, Peru; 3 Fundación Oftalmológica Nacional, Bogotá 110221, Colombia; fjrodriguez@fon.org.co; 4 Department of Ophthalmology, Illinois Eye and Ear Infirmary, School of Medicine, University of Illinois Chicago, Chicago, IL 60612, USA; 5 Drs. Berrocal and Associates, San Juan 00907, Puerto Rico; mariahberrocal@hotmail.com

**Keywords:** diabetic retinopathy, diabetic macular edema, retinal neovascularization, retinal angiogenesis, angiopoietins, Tie2, angiopoietin–tie pathway, faricimab

## Abstract

Complications from diabetic retinopathy such as diabetic macular edema (DME) and proliferative diabetic retinopathy (PDR) constitute leading causes of preventable vision loss in working-age patients. Since vascular endothelial growth factor (VEGF) plays a major role in the pathogenesis of these complications, VEGF inhibitors have been the cornerstone of their treatment. Anti-VEGF monotherapy is an effective but burdensome treatment for DME. However, due to the intensive and burdensome treatment, most patients in routine clinical practice are undertreated, and therefore, their outcomes are compromised. Even in adequately treated patients, persistent DME is reported anywhere from 30% to 60% depending on the drug used. PDR is currently treated by anti-VEGF, panretinal photocoagulation (PRP) or a combination of both. Similarly, a number of eyes, despite these treatments, continue to progress to tractional retinal detachment and vitreous hemorrhage. Clearly there are other molecular pathways other than VEGF involved in the pathogenesis of DME and PDR. One of these pathways is the angiopoietin–Tie signaling pathway. Angiopoietin 1 (Ang1) plays a major role in maintaining vascular quiescence and stability. It acts as a molecular brake against vascular destabilization and inflammation that is usually promoted by angiopoietin 2 (Ang2). Several pathological conditions including chronic hyperglycemia lead to Ang2 upregulation. Recent regulatory approval of the bi-specific antibody, faricimab, may improve long term outcomes in DME. It targets both the Ang/Tie and VEGF pathways. The YOSEMITE and RHINE were multicenter, double-masked, randomized non-inferiority phase 3 clinical trials that compared faricimab to aflibercept in eyes with center-involved DME. At 12 months of follow-up, faricimab demonstrated non-inferior vision gains, improved anatomic outcomes and a potential for extended dosing when compared to aflibercept. The 2-year results of the YOSEMITE and RHINE trials demonstrated that the anatomic and functional results obtained at the 1 year follow-up were maintained. Short term outcomes of previously treated and treatment-naive eyes with DME that were treated with faricimab during routine clinical practice suggest a beneficial effect of faricimab over other agents. Targeting of Ang2 has been reported by several other means including VE-PTP inhibitors, integrin binding peptide and surrobodies.

## 1. Introduction

The global prevalence of diabetes mellitus (DM) has reached epidemic proportions without an end in sight. According to estimates of the International Diabetes Federation (IDF), in 2021, there were 537 million people affected by DM worldwide. By 2030, this number is estimated to increase to 643 million people and to 783 million by 2040. The vast majority of this increase will occur in low- and middle-income countries. Furthermore, 240 million people with DM are currently undiagnosed. Most of these undiagnosed individuals also live in low- and middle-income countries [1]. All of these individuals will be at risk of developing diabetic retinopathy (DR).

If left untreated, patients with DR can suffer severe visual loss [2]. In developed countries DR constitutes the leading cause of blindness in the working-age population and has a considerable economic impact on society particularly on healthcare systems [3,4,5,6,7].

DR is a progressive condition characterized by microvascular alterations that lead to retinal ischemia, an increase in retinal vasopermeability, retinal neovascularization and macular edema [8,9]. Diabetic macular edema (DME) is characterized by an excessive vascular permeability that leads to an extravasation of plasma constituents and accumulation of extracellular fluid in the inner retina [10]. Proliferative diabetic retinopathy (PDR) is characterized by progressive retinal ischemia which leads to retinal neovascularization and tractional retinal detachment [11].

The molecular pathways involved in the pathogenesis of DR have been elucidated in part. Several cytokines and growth factors have been implicated in the development of DR [12]. Of these, vascular endothelial growth factor (VEGF) has been one of the most studied and appears to be one of the most important ones [13]. Despite the demonstrated efficacy of anti-VEGF drugs in the treatment of DME in randomized clinical trials [14,15,16], studies of routine clinical practice reveal widespread undertreatment which compromises the visual outcomes [17,18]. Even in adequately treated patients, persistent DME is reported anywhere from 30% to 60% depending on the drug used [19]. Since the discovery of the angiopoietins (Ang) and their respective tyrosine kinase with immunoglobulin and epidermal growth factor homology domains (Tie) receptors in the 1990’s [20,21,22], mounting evidence has implicated the angiopoietin–Tie signaling pathway in the pathogenesis of DR [23,24,25,26,27,28]. Targeting the Tie signaling pathway may improve the outcomes of patients with DR. Recently, faricimab, a heterodimeric bispecific monoclonal antibody that targets both VEGF and the angiopoietin–Tie signaling pathways, has been approved by different regulatory agencies across the globe and introduced into clinical practice [29]. This major review will focus on the Ang–Tie signaling pathway as it relates to DR.

## 2. The Angiopoietins and Tie Signaling Pathway

### 2.1. The Angiopoietins

The angiopoietins together with the members of the VEGF family are the only known growth factors that have a high specificity for endothelial cells (EC) [30]. The angiopoietins consist of a family of secreted glycoprotein growth factors. They are multimeric ligands that act primarily through the Tie2 receptor. The four different angiopoietins (Ang1-4) consist of very similar modular structures [31,32]. There is a fibrinogen-like receptor binding domain (FReD) at the carboxy terminal, a linker region, a dimerization motif and an amino terminal superclustering coiled-coil motif. The FReD consists of three subdomains: A, B and P [33].

Ang1 is produced and secreted primarily by pericytes and platelets. Inside the eye, Ang1 is expressed in the neural crest derived tissues such as the trabecular meshwork (TM), the sclera, the choroid and retinal pericytes [34,35,36,37,38]. As Ang1 is secreted, it predominantly exists as higher-order multimers [32,39]. Tie2 serves as the native receptor of Ang1. Ang1 tetramers are the minimal size required for Tie2 activation in EC. Ang1 monomers and Ang1 dimers inhibit Tie2 receptor activation [31]. Typically, Ang1 tetramers bind to Tie2 leading to Tie2 autophosphorylation which initiates an intracellular signaling cascade [20]. Under certain circumstances, Ang1 may signal through Tie1 and integrins [40,41,42].

EC produce and store Ang2 in Weibel–Palade bodies under quiescent conditions. Ang2 is released via Weibel–Palade bodies exocytosis when the EC is activated by hyperglycemia, inflammation, hypoxia or cancer [23,24,43,44,45,46,47,48]. The intracellular Ang2 stores have a long half-life and recover rapidly following release [49]. Depending on the cellular context, Ang2 can act as an antagonist or as a weak Tie2 agonist [50].

Chromosomal localization studies of all the angiopoietins in human and mice demonstrate that Ang3 and Ang4 represent the mouse and human counterparts of the same gene locus [51]. In comparison to Ang1 and Ang2, the biological functions of both Ang3 and Ang4 are poorly defined. Ang4 is less oligomerized and exhibits a lower affinity to Tie2 than Ang1 and Ang2. Similar to Ang1, and unlike Ang2, Ang4 induces Tie2 autophosphorylation upon binding to Tie2 [52]. Ang4 appears to play a role in venous development of the mouse retina and in aqueous humor drainage [52,53].

### 2.2. TIE Receptors

Receptor tyrosine kinases (RTK) activation leads to intracellular signal transduction. The only known EC-specific RTKs are the VEGF receptors and the Tie receptors [30]. Typically, RTKs are located on the surface of the cell and have intracellular and extracellular domains. When a ligand binds to the RTK via its extracellular domain, the RTK undergoes dimerization and autophosphorylation of its tyrosine residues on the intracellular domain. This sets into motion a cascade of cellular responses [54,55].

The intracellular tyrosine kinase domain of both Tie1 and Tie2 consists of a kinase insert and a transmembrane helix [56,57]. The extracellular domain contains a membrane-distal ligand binding region (LBR) at the amino terminal which is linked via a coiled coil region to a C-terminal receptor binding fibrinogen-related domain (FrED) [31]. The LBR contains two immunoglobulin (IgG) domains followed by three epidermal growth factor (EGF) repeats and a third IgG domain [58,59]. FrED consists of three fibronectin type III (FNIII) repeats in the extracellular region next to the transmembrane catalytic tyrosine kinase domain found intracellularly at the carboxyl terminal [21,56,57,60,61,62,63,64,65].

Tie1 is mostly expressed in some hematopoietic cells and in both the developing and mature EC of blood and lymphatic vessels [65]. Tie1 controls angiopoietin binding to Tie2, determining the context dependence of the Ang2 interaction with Tie2, and serving as a modulator of Tie2 transduction [58,66,67]. Tie2 is ubiquitously expressed in the endothelium of all organs and tissues, particularly in angioblasts and in sprouting blood vessels throughout development [63]. Tie2 is usually highly expressed in healing skin wounds and in the quiescent vasculature of adult tissues. Curiously, the Tie2 immunoprecipitated from these tissues is tyrosine phosphorylated indicating active downstream signaling in the adult quiescent vasculature [68].

### 2.3. Vascular Endothelial-Protein Tyrosine Phosphatase (VE-PTP)

Human protein tyrosine phosphatase beta (HPTPb) is the human ortholog of the mouse VE-PTP where they both share 92% sequence identity [69]. It is an EC-specific receptor protein tyrosine phosphatase. Its substrates include Tie2, VE-cadherin, VEGFR2 and the GTPase exchange factor FGD5 [70]. VEGFR2 and VE-PTP are found in close proximity to each other [71]. VE-PTP forms a complex with VEGFR-2 during normal quiescent physiological conditions. When VEGF is upregulated and binds to VEGFR-2, the VE-PTP-VEGFR-2 complex separates and permits dimerization of the VEGFR2 and downstream signaling leading to increased vascular permeability. With time, the VE-PTP and VEGFR2 complex resume causing dephosphorylation and de-activation of VEGFR2. Inhibition of VE-PTP leads to an increased VEGFR2 activity and signaling suggesting that VEGFR2 serves as a substrate for VE-PTP [71].

VE-PTP is the main and most downstream negative regulator of Tie2. It binds to Tie2 and acts by dephosphorylating Tie2 [72,73]. VE-PTP modulates and regulates Tie2 signaling by forming hetero-oligomers with Tie2 and hydrolyzing critical phosphotyrosines [74,75]. In EC, VE-PTP physically binds to Tie2 via its cytoplasmic phosphatase domain. This interaction leads to dephosphorylation and attenuation of Tie2 signaling [69,72,74].

VE-cadherin is an EC-specific cell adhesion molecule that plays a central role in the maintenance of the inter-endothelial cell contacts. It is anchored to the cellular actin cytoskeleton via the catenins, which are intracellular proteins. VE-cadherin is constitutively dephosphorylated. Phosphorylation of VE-cadherin, b-catenin and p120-catenin causes vascular instability which is characterized by vascular permeability and leukocyte extravasation. Tyrosine phosphorylation of the Y685 and the Y731 tyrosine residues of VE-cadherin is implicated in the regulation of endothelial permeability and the transmigration of leukocytes [76,77,78,79,80]. Blocking antibodies to the VE-cadherin adhesive function causes destabilization of the endothelial junctions [81,82,83]. In the EC, VE-PTP associates with VE-cadherin via their ectodomains, leading to dephosphorylation of VE-cadherin and enhancement of the barrier function of VE-cadherin [84]. Dissociation of VE-PTP from VE-cadherin leads to destabilization of endothelial junctions. Inhibition of VE-PTP causes a reduction in the adherence function of VE-cadherin [85].

By virtue of its effects on its substrates, VE-PTP regulates developmental and tumor angiogenesis, controls vascular permeability and modulates homeostasis in inflammation [75]. The effects of VE-PTP on vascular permeability are context-dependent. On the one hand, inhibition of VE-PTP leads to Tie2 activation and stabilization of endothelial junctions. On the other hand, inhibition of VE-PTP leads to the loss of the adherence function of VE-cadherin and increased VEGFR2 activity. In general, the effect over Tie2 is dominant over the effect of VE-cadherin [86].

VE-PTP is expressed during embryogenesis and adulthood suggesting a role in normal vascular homeostasis, vascular remodeling, endothelial junction integrity and vascular permeability [85,86,87,88]. Mice with genetically knocked-out VE-PTP undergo vasculogenesis but die during embryonic development due to vascular enlargement in extra-embryonic tissue plus defects in both angiogenesis and vascular remodeling [73,87,89]. VE-PTP genetic disruption can be mimicked by antibodies against the extracellular portion of VE-PTP [73]. These antibodies cause dissociation of VE-PTP from Tie2 by triggering selective endocytosis of only the Tie2-associated protein fraction of VE-PTP. In contrast, the VE-cadherin-associated protein fraction of VE-PTP remained intact. This results in activation of Tie2 which leads to Erk 1/2 activation resulting in EC proliferation and enlargement of vascular structures. VE-PTP may serve as a molecular balance to counteract Tie2-induced EC proliferation. Vessel size and vascular development may be controlled in this fashion [73]. VE-PTP/HPTPb is upregulated during hypoxia and impairs Ang1-induced Tie2 phosphorylation [72].

The importance of the Ang/Tie signaling on vascular development and homeostasis is underscored by the expression pattern found during embryonic development and the embryonic lethality of genetic deletions of Ang1, Tie1 and Tie2 in animal models [90,91].

### 2.4. Ang/Tie Signaling Pathway in the Retina

Once the adult vasculature has been established, the ECs remain quiescent with the exception of the female reproductive system [92]. Under normal physiological and homeostatic conditions, Ang1 is constitutively expressed by retinal perivascular cells such as pericytes lining adult blood vessels in the ganglion cell layer (GCL) and the retinal inner nuclear layer (INL) [20,93]. Ang1 plays a major role in maintaining vascular quiescence and stability. It acts as a molecular brake against vascular destabilization and inflammation that is usually promoted by Ang2 [94]. Ang1 levels outnumber Ang2 since Ang1 is basally secreted under normal physiological conditions in adults, whereas Ang2 levels are low and found only at sites of vascular remodeling [22,49,95,96,97,98].

In vitro studies have shown that Ang1 diminishes the increased permeability caused by common vasopermeability mediators such as histamine, bradykinin, VEGF-A, serotonin and PAF, among others. The anti-permeability effects of Ang1 are mediated via the strengthening of the cellular junctions through Rap1, a small GTPase. Ang1 stimulation leads to Rap1 activation. Rap1 is an essential signaling intermediate that mediates the functions of VE-cadherin and is also involved in Ang1-induced cell–cell junction stabilization. Rap1 activation is necessary for the anti-permeability effects of Ang1 [99].

Ang1 binding to the extracellular domain of Tie2 results in Tie2 autophosphorylation which activates a multitude of downstream intracellular signaling pathways including the phosphatidyl inositol 3-kinase (PI3k), protein kinase B (AkT), extracellular signal regulated kinase (ERK) 1/2, p38 mitogen activated protein kinase (MAPK) and stress-activated protein kinase (SAPK)/ Jun amino-terminal kinase (JNK) pathways [68,94,100,101,102,103,104,105,106,107,108].

Tie2 signaling may elicit paradoxical responses. On the one hand, Tie2 signaling maintains the quiescence and stability of mature vessels [94]. On the other hand, Tie2 signaling also plays a role in physiological and pathological angiogenesis [68,100,101,102,103]. The distinct localization of Tie2 in the presence or absence of EC-EC contact determines the specificity of downstream signaling of Tie2 [104,105]. In the absence of EC-EC contacts, Tie2 is anchored to the ECM by Ang1. The ERK 1/2 pathway is preferentially activated [106]. In the presence of VEGF, the Ang1-induced ERK1/2 phosphorylation is augmented [107]. If EC-EC contacts are present, then Ang1 induces trans-association of Tie2 which preferentially activates the PI3k-AkT pathway [106]. Akt is a major angiogenic mediator downstream of the Ang1/Tie2 signaling pathway [108].

In the normal retina, Ang2 is expressed in EC, horizontal cells, cells in the GCL, Müller cells and INL [109,110,111,112]. The EC of mature quiescent vessels are the primary source of Ang2 [43,49,110,113]. von Willebrand factor polymerization within EC form storage organelles called Weibel–Palade bodies, which serve as storage sites of Ang2 and P-selectin. However, the storage of P-selectin and Ang2 are mutually exclusive [49,114,115]. When released, Ang2 acts in an autocrine manner activating or inhibiting Tie2 on the ECs depending on the cellular context [22,50,110,116,117,118]. Tie1 holds the key for the context dependence of Ang2 [58,66,67]. Ang2 acts as a weak Tie2 agonist in the presence of Tie1 but in its absence functions as an antagonist even at low concentrations [119].

Gene silencing studies using siRNA against HPTPb demonstrated an increased Tie2 phosphorylation by both Ang1 and Ang 2. However, the effects were different depending on the ligand. There was increased activation of Akt and EC survival following Ang1 exposure. In contrast, Ang2 did not elicit an increased EC survival. These findings suggest that HPTPb plays an important role in regulating Ang1-Tie2 signaling [72].

## 3. Molecular Mechanisms and Pathophysiology of Diabetic Retinopathy

### 3.1. Effects of Hyperglycemia and Ang–Tie Signaling

At the molecular level, several animal models implicate the angiopoietins and Tie2 in the pathogenesis of DR [23,24,25,26,27,28]. Hyperglycemia induces Ang2 upregulation in both retinal EC in vitro and in diabetic animals prior to any morphological changes in the retinal capillaries [28,48]. Ang2 upregulation occurs via post-translational modification of a co-regulator protein that results in increasing ANGPT2 expression [48,120,121,122]. Under high glucose conditions, during glycolysis, triose phosphates form a highly reactive dicarbonyl degradation product called methylglyoxal. Methylglyoxal covalently modifies intracellular proteins by forming stable adducts with their arginine residues. One of these proteins is the co-repressor protein mSin3A which normally forms a complex with the transcription factor Sp3. The mSin3A-Sp3 complex normally inhibits Ang2 transcription by binding to a glucose-responsive GC-box in the Ang2 promoter. Methylglyoxal modification of mSin3A causes an increased recruitment of O-GlcNAc transferase to the mSin3A-Sp3 complex which leads to an augmented modification of Sp3 by O-linked N-acetylglucosamine. This modification of Sp3 causes decreased binding of the mSin3A-Sp3 complex to the Ang2 promoter which results in increased transcription of Ang2 [121].

In addition, several biochemical alterations caused by chronic hyperglycemia, including production of advanced glycation end (AGE) products, activation of protein kinase C, an increased hexosamine pathway flux and an increased polyol pathway flux, upregulate Ang2 altering the delicate balance between Ang1 and Ang2. This leads to an increased vascular permeability, followed by EC loss, vascular occlusion and retinal neovascularization [8,9,26,123,124,125,126,127,128].

An increased flux through the hexosamine pathway causes glucose to be metabolized into UDP-N-acetylglucosamine. The cellular supply of nucleoside triphosphate is dependent on nucleoside diphosphate kinases (NDPK) which are therefore critical for certain cellular activities such as cellular proliferation, cellular differentiation, cellular adhesion, cellular molecular transport and apoptosis. NDPK isoform B (NDPKB) catalyzes the formation of UTP from UDP and ATP. NDPKB contributes to the BRB by regulating the distribution of adherens junction proteins and caveolins in EC [129,130]. NDPKB deficiency causes Ang2 up-regulation in retinal EC [131].

The renin–angiotensin system (RAS) is also involved in the pathogenesis of diabetic retinopathy [132,133]. An in vitro study showed that angiotensin II induced VEGF production in pericytes. This VEGF can then be released by the pericytes and stimulate the retinal EC in a paracrine manner [134]. The renin–angiotensin system may modulate the angiopoietins as well [135]. Even though VEGF upregulates Ang2, Ang2 upregulation by angiotensin II is independent of VEGF. In bovine retinal EC, angiotensin II binds to the ATI receptor which in turn activates the PKC and MAPK pathways leading to an increase in the rate of Ang2 mRNA transcription and Ang2 protein synthesis. Angiotensin II does not have an effect on Ang1 [135].

AGEs during chronic hyperglycemia may modify Tie2 and upregulate both VEGF and Ang2 mRNA in EC [25,122]. Glycation of Tie2 by AGE products inhibits Ang1-induced Tie2 autophosphorylation and Akt downstream signaling. Inhibition of AGEs by aminoguanidine rescues Ang1-induced Tie2 autophosphorylation. Glycation of Tie2 amino acid residues can alter its structure affecting its binding affinity to Ang1. Alternatively, glycation may directly reduce tyrosine kinase activity.

**Figure 1:**
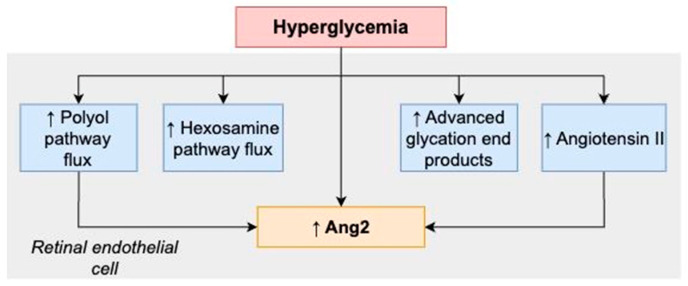
Summary of the relationship between hyperglycemia and Ang2.

The effects of hyperglycemia on Ang–Tie signaling are summarized in Figure 1.

### 3.2. Ang–Tie Signaling and Blood Retinal Barrier

An early pathological change observed in DM is leukocyte adhesion to the retinal vasculature via ICAM-1. This adherence causes capillary occlusion, EC damage and death which leads to the breakdown of the blood retinal barrier (BRB), an important hallmark of DM [127,128]. Ang1 inhibits phosphorylation of PECAM-1 and VE-cadherin. Leukocyte diapedesis is inhibited by Ang1/Tie2 signaling [124]. During inflammation, Ang1 inhibits leukocyte recruitment, leukocyte adhesion in the retinal vessels and prevents retinal EC injury enhancing its barrier function [26,124,125,126].

Another effect of chronic hyperglycemia is IL-6 upregulation. In patients with DME, intravitreal levels of IL-6 and VEGF are elevated when compared to non-diabetic patients. The intravitreal IL-6 level was significantly correlated with the intravitreal level of VEGF. Both of these were correlated with the severity of DME [136]. IL-6 induces STAT-3 activation and phosphorylation via JAK activation [137]. VEGF expression is upregulated by activated STAT3 which leads to downregulation of the expression of endothelial tight junction proteins ZO-1 and occludin, leading to an increased vascular permeability [138]. In animal models, Ang1 can prevent and reverse these diabetic changes in the retinal vasculature [26]. Ang1 reduced the breakdown of the BRB in a dose-dependent manner by attenuating IL-6 induced vascular permeability by dissociating the tyrosine phosphatase SHP-1 from Tie2. SHP-1 can then bind to JAK1, JAK2 and STAT3 inhibiting IL-6-induced phosphorylation of STAT3 downregulating EC VEGF secretion [26,139].

Ang2 is an important regulator of the BRB in DM through its actions on pericytes and astrocytes [23,24,140]. In diabetic mice, pericyte loss occurs at a slightly later stage than astrocyte loss. In streptozotocin-induced diabetic mice, retinal astrocytes die early in the course of the disease. In these animals, retinal vascular leakage increased with increasing astrocyte loss. Ang2 inhibition blunted retinal astrocyte death and retinal vascular leakage [24]. Mice with an Ang2 heterozygous deficiency prevents pericyte loss and subsequent formation of acellular capillaries [48]. In vitro models show that under high glucose conditions, the ανβ5 integrin is highly expressed in astrocytes. In contrast, Tie2 is not expressed in astrocytes under high-glucose conditions. Astrocyte apoptosis is mediated via the activation of the ανβ5 integrin/GSK-3β/β-catenin pathway [23,24]. Ang2 binds to the ανβ5 integrin leading to dephosphorylation of GSK-3β and phosphorylation of β-catenin resulting in β-catenin degradation causing astrocyte apoptosis. Inhibition of both GSK-3β and the ανβ5 integrin attenuates Ang2-induced astrocyte apoptosis. Intravitreal injection of an anti-ανβ5 integrin antibody diminished astrocyte death in streptozotocin-induced diabetic mice [24].

Pericyte loss is often recognized as the first morphological change in diabetic retinas [141]. Since pericytes are crucially involved in promoting retinal EC survival, their loss leads to EC death causing microaneurysm formation, acellular capillaries and capillary nonperfusion. Retinal neovascularization and microaneurysm formation occurs at the sites of pericyte and astrocyte loss [95,142]. Since hyperglycemia induces Ang2 upregulation, it follows that Ang2 may be responsible for pericyte loss in diabetic patients [48,120,121,143]. In non-diabetic animals, intravitreal Ang2 and intraocular Ang2 overexpression causes pericyte loss and acellular retinal capillaries [48,140,143,144].

Experimental models have shown that pericytes are lost by two different mechanisms [23,144]. In a diabetic rat model, an upsurge of Ang2 preceded the onset of pericyte apoptosis and loss. This pericyte loss was abolished in hemizygous Ang2 LacZ knock-in mice that expressed significantly less Ang2. Under high-glucose conditions, α3β1 integrin but not Tie2 is upregulated in pericytes. Ang2 induces pericyte apoptosis via α3β1 integrin signaling through p53. Inhibition of a3b1 integrin attenuates Ang2-induced pericyte apoptosis [23]. In another experimental model of diabetic retinopathy, pericyte loss was the result of pericyte migration [144].

Ang2 acts in concert with VEGF to facilitate the breakdown of the BRB, EC migration and proliferation [101]. Ang2 plays an important role in the increased vasopermeability observed in diabetic retinopathy by VE-cadherin phosphorylation and an increased vasopermeability [28]. An in vitro study demonstrated that under normal conditions, tight junctions are present between porcine retinal EC. After incubation with Ang2 and VEGF, the tight junctions disappeared which led to an increased permeability. VEGF was twice as potent as Ang2 in inducing permeability, but the combination of both VEGF and Ang2 led to three times as much permeability as VEGF alone [27].

**Figure 2:**
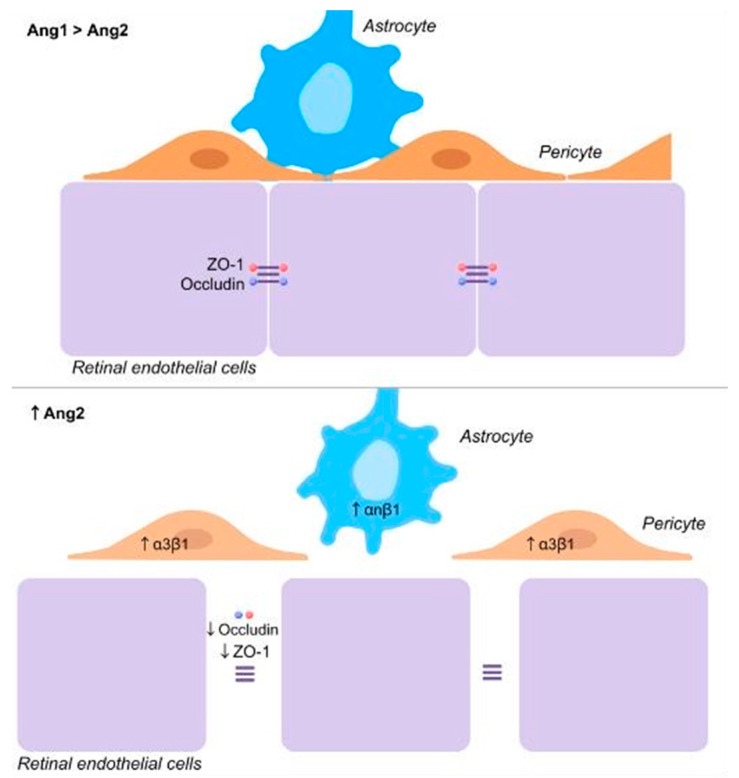
Summary of the effects of Ang1 and Ang2 on the blood–retinal barrier.

Figure 2 summarizes the effect of Ang1 and Ang2 on the BRB.

### 3.3. Vascular Regression and Ang–Tie Signaling

Ang2 is crucial for initiating vascular regression in diabetic retinopathy [48,144,145,146]. The presence or absence of VEGF determines the biological effect of Ang2. In the absence of VEGF, Ang2 causes vessel regression, whereas in its presence, Ang2 causes angiogenesis [101,147,148,149,150,151,152,153]. In the cornea micropocket assay of the neovascularization model, Ang2, like Ang1, did not promote neovascularization. Co-administration of Ang2 and VEGF led to an increase in vessel circumferential extent and vessel length. Furthermore, EC at the leading tip of capillaries were seen migrating toward the Ang2 and VEGF. Increasing VEGF levels also seem to upregulate Ang2 levels, further stimulating angiogenesis.

Endothelial progenitor cells are typically mobilized from the bone marrow to sites of vascular injury to attempt to repair the injured tissue. In a rat model of diabetic retinopathy, the levels of circulating endothelial progenitor cells were significantly less in diabetic animals compared to non-diabetic animals. Simvastatin increased circulating endothelial progenitor cells which led to a decrease in the incidence and progression of diabetic retinopathy in rats [154]. Simvastatin suppressed superoxide formation and decreased expression of erythropoietin, VEGF, Ang1 and Ang2 in the retina of these diabetic animals [154,155]. Simvastatin may modify the intravitreal levels of Ang2 in diabetic patients requiring vitrectomy [156]. In a prospective cross sectional case–control study, 14 patients on simvastatin were compared to 50 patients who did not take simvastatin. Patients that were treated with simvastatin exhibited significantly lower intravitreal levels of Ang2, VEGF and MMP-9. Adding acetylsalicylic acid further lowered the intravitreal levels [156].

### 3.4. Retinal Neovascularization and Ang–Tie Signaling

Once the adult vasculature has been established, the ECs remain quiescent with the exception of the female reproductive system [92]. All other angiogenic activity during adulthood occurs in response to injury or disease. Many ocular pathological conditions such as RVO, PDR, sickle cell retinopathy and ROP are characterized by retinal ischemia. Ischemia leads to hypoxia which is the major driver of pathological angiogenesis. The oxygen induced retinopathy (OIR) animal models serve as prototypes for these ischemic retinopathies [110,157]. Several OIR models have shown that Ang2 inhibition inhibits retinal neovascularization. In a mouse OIR model, both VEGF and hypoxia upregulated Ang2 mRNA expression as well as protein synthesis [158,159,160].

### 3.5. Diabetic Patients and Ang–Tie Signaling

Diabetic patients experience higher serum and plasma Ang2 levels than non-diabetic individuals regardless of the presence or absence of cardiovascular disease. Plasma and serum Ang2 levels were directly correlated with plasma and serum VEGF levels and HbA1c [161,162,163]. Interestingly, Ang2 levels fell as a result of panretinal photocoagulation. In contrast, the Ang1 levels were not uniform. Some studies reported non-elevated levels of Ang1, but in another study, Ang1 levels were elevated [164]. Single nucleotide polymorphisms may be associated with the risk of developing diabetic retinopathy. The rs2442598A allele in the ANGPT-2 gene is associated with an increased risk for diabetic retinopathy in Brazilian patients with type 1 DM [165]. Intravitreal levels of Ang2 are also elevated in eyes with DME and PDR [166,167,168]. A recent systematic review and meta-analysis reported that intravitreal levels of Ang2 are elevated in eyes with PDR when compared to healthy non-diabetic eyes [169]. A retrospective case–control study compared the vitreous levels of Ang2 in eyes with PDR to eyes with no diabetic ocular disease. There was an increased intravitreal concentration of both Ang2 and VEGF in eyes with PDR. Eyes with active PDR exhibited higher Ang2 levels than inactive PDR. Furthermore, the levels of Ang2 correlated with those of VEGF [170]. Another cross sectional study reported that in eyes with PDR, Ang1, Ang2, VEGF-A, PlGF, Il-8 and Il1b were all elevated when compared to non-diabetic eyes [171]. In twelve patients with PDR, epiretinal membranes were surgically removed and analyzed with immunohistochemistry. Both Ang2 and Tie2 were significantly upregulated in these specimens when compared to specimens removed from eyes with a primary epiretinal membrane [172].

## 4. Targeting the Ang–Tie Signaling Pathway in DR

Recognition of the value of therapeutic targeting of the Ang/Tie signaling has led to the regulatory approval and commercialization of faricimab in many countries. Several other promising strategies that target the Ang–Tie pathway have been explored as well. Targeting of Ang2 has been reported by several other means, including VE-PTP inhibitors, integrin binding peptide and surrobodies.

### 4.1. Combination Therapy of Anti-Ang2 and Anti-VEGF

- Faricimab (RO6867461, RG7716, Roche)

Faricimab-svoa is a heterodimeric bispecific monoclonal antibody developed using CrossMab technology that targets both VEGF-A and Ang2 [173]. CrossMab technology enables correct antibody light chain association with their respective heavy chain and thus enforcing specific heterodimerization of two different antigen binding domains [166,174]. Since the angiopoietins have structural similarities, it is important that the monoclonal antibody binds only to Ang2 and not Ang1 [175]. The fragment crystallizable (Fc) region of faricimab has been modified so that it does not bind to the FcgR and FcRn receptors. The FcgR mediates effector functions such as antibody-dependent cytotoxicity, antibody-dependent cell phagocytosis and complement-dependent cytotoxicity. The modifications in faricimab abolish these effector functions [166]. The FcRn receptor recycles IgG, so the modifications made in faricimab reduces its systemic half-life without compromising its intravitreal half-life when compared to a wild-type IgG. As a result, faricimab has less potential for intraocular inflammation, a reduced systemic absorption and a faster systemic clearance [166,176].

The BOULEVARD study was a multicenter, double-masked, randomized phase 2 clinical trial that compared 0.3 mg ranibizumab to 6 mg faricimab or 1.5 mg faricimab in eyes with previously treated and treatment-naive DME [177]. All eyes received 6 monthly injections according to the randomization scheme. At 24 weeks of follow-up, 6 mg faricimab had superior visual gains over ranibizumab. In addition, central subfield thickness (CST), diabetic retinopathy severity score (DRSS) and durability were all improved with faricimab compared to ranibizumab. An exploratory analysis suggested that in previously treated eyes, the BCVA and DRSS gains were similar to the ones obtained with ranibizumab. In contrast, the CST reduction and durability were greater with faricimab than ranibizumab. More than 90% of eyes showed no disease reactivation 8 weeks after the last faricimab injection suggesting an increased durability of faricimab in these eyes. This formed the basis of the personalized treatment interval (PTI) in the YOSEMITE and RHINE pivotal trials [168]. Limitations of this study include its short treatment and off-treatment observation periods of 20 and 16 weeks, respectively [177].

The YOSEMITE and RHINE were multicenter, double-masked, randomized non-inferiority phase 3 clinical trials that compared faricimab to aflibercept in eyes with center-involved DME. Previously treated eyes were capped at 25% of the enrollment [168,178]. Patients were randomized to 2 mg of aflibercept injected every 2 months after a loading dose of 5 monthly injections, 6 mg of faricimab injected every 2 months following a loading dose of 6 monthly injections and a PTI arm of 6mg of faricimab after 4 monthly loading injections [168]. The PTI arm was modeled after a treat and extend regimen that is commonly used in clinical practice. In the PTI arm, the monthly dosing was maintained until the central subfield thickness (CST) decreased to <325 µm. Once this was achieved, the treatment intervals were extended to every 8 weeks. Depending on the visual acuity and CST gains and losses, the treatment interval could be extended up to 16 weeks or reduced to 4 weeks.

At 12 months of follow-up, faricimab demonstrated non-inferior vision gains, improved anatomic outcomes and a potential for extended dosing when compared to aflibercept [178]. The primary endpoint was the mean change in BCVA score from baseline to weeks 48, 52 and 56. The faricimab PTI arms gained an average of 11.6 and 10.8 letters compared to 11.8 and 10.7 letters in the faricimab every other month arm and 10.9 letters in the aflibercept arms. Secondary endpoints included the percentage of patients with a central subfield thickness < 325 µm at weeks 48,52 and 56; percentage of patients with absence of subretinal fluid at week 52; percentage of intraretinal fluid at week 5; and the percentage of patients in the faricimab arms on different treatment intervals at week 48. The PTI arms demonstrated faricimab’s strong durability, 50% of eyes achieved 16-week dosing and an additional 20% achieved 12-week dosing. In the YOSEMITE/RHINE trials, 144 Asian patients were enrolled [179]. The 12-month anatomical, functional, durability and safety outcomes of Asian and non-Asian patients were comparable [179].

The 2-year results of the YOSEMITE and RHINE trials demonstrated that the anatomic and functional results obtained at the 1 year follow-up were maintained [180]. At 2 years, the mean BCVA change from baseline was comparable in all three treatment arms in both trials. The faricimab every other month arm gained 10.7 and 10.9 letters compared to 10.7 and 10.1 letters in the faricimab PTI arm and 11.4 and 9.4 in the aflibercept arm. The mean change in central subfield thickness was −209 µm, −201 µm and −191 µm for the faricimab every other month, faricimab PTI and aflibercept arms, respectively. Faricimab was a better drying agent than aflibercept as evidenced by the proportion of eyes with an absence of DME and intraretinal fluid. At 2 years, 88–92% of eyes in the faricimab every other month arm compared to 81–86% in the faricimab PTI arm and 79–83% in the aflibercept arms had an absence of DME. At 2 years, 57–63% of eyes in the faricimab every other month arm compared to 44–48% in the faricimab PTI arm and 36–41% in the aflibercept arms had an absence of intraretinal fluid. At 2 years, the proportion of eyes with a ≥2 step improvement in the DRSS was 52.4% in the faricimab every other month arm, 43.5% in the faricimab PTI arm and 43% in the aflibercept arm. These results were achieved with a median of 10–11 injections in the faricimab PTI group, 15 injections in the faricimab every other month group and 14 injections in the aflibercept group. Furthermore, eyes in the faricimab PTI group exhibited an improved durability with >60% achieving 16-week dosing and almost 80% achieved a dosing interval of at least every 12 weeks. Only 4% of eyes in the faricimab arm required monthly dosing.

A systematic literature review identified 26 randomized clinical trials published before August 2021 that were used to conduct a network meta-analysis to compare faricimab treatment and extend to intravitreal aflibercept, ranibizumab, bevacizumab, dexamethasone implant and laser treatment [181]. Network meta-analysis allow treatments that are not compared directly within randomized clinical trials to be compared indirectly. In this network meta-analysis at 12 months of follow-up, treatment and extended treatment regimen for faricimab demonstrated superior retinal drying which was achieved with fewer injections when compared to other flexible dosing regimens. In addition, visual outcomes at 12 months were superior with the treat and extend faricimab when compared to bevacizumab and ranibizumab [181]. Another systematic review and meta-analysis of three randomized clinical trials from 2013 to 2023 that included 2120 patients with DME concluded that faricimab was a better drying agent as manifested by a greater reduction in CST compared to other anti-VEGF agents [182]. This reduction was achieved with a lower number of injections. However, there were no differences in terms of changes in BCVA or gain in 15 letters [182].

Short term outcomes of eyes with DME treated with faricimab during routine clinical practice have been reported in both previously treated and treatment-naive eyes [183,184,185,186,187,188]. A small retrospective case–control study of 51 eyes with persistent DME despite aflibercept treatment compared the anatomic and functional outcomes of eyes switched to faricimab to those who continued aflibercept. Patients that were switched to faricimab received three consecutive monthly injections, and those who remained on aflibercept also received three consecutive monthly injections. One month after the last injection, 37.5% (9/24) of faricimab-treated eyes and 3.7% (1/27) of aflibercept-treated eyes had an OCT with no retinal fluid and a CMT ≤ 300 µm. There were 41.7% of faricimab-treated eyes and 11.1% of aflibercept eyes that gained ≥ 2 lines of BCVA [184]. In another retrospective study, 51 eyes with recalcitrant DME despite aflibercept therapy were switched to faricimab. Faricimab-treated patients underwent a loading dose of three consecutive monthly injections and were then assessed for retinal fluid. If there was no more retinal fluid, the eyes were treated on a treat and extend protocol. At the end of 12 months, 39.2% of eyes achieved a dry macula and a treatment interval of ≥ 8 weeks. The average reduction in CMT and the average improvement in BCVA were statistically significant [183]. A retrospective study of 18 eyes that had residual IRF or SRF or had a treatment interval less than 8 weeks despite ranibizumab or aflibercept treatment were switched to as-needed faricimab. At 4 months, the treatment interval was increased from 5.8 to 10.8 weeks following the switch to faricimab. In 44% of eyes, the treatment interval was extended to ≥12 weeks. In 16.7% of eyes, a monthly treatment interval was required after the switch. Despite the switch to faricimab, the BCVA and the CMT were not significantly different [185]. Another retrospective Japanese study reported on 21 eyes that had a minimum follow-up of one month following faricimab treatment. In this cohort, there were 14 treatment-naive eyes and 7 previously treated eyes. After a mean follow-up of 5.5 months, eyes received a mean of 1.6 faricimab injections. Six (29%) eyes (four treatment-naive and two previously treated) were switched to alternative treatments such as aflibercept, brolucizumab, sub-Tenon triamcinolone and pars plana vitrectomy for various reasons including lack or insufficient response to faricimab and faricimab side effects [187]. Another study prospectively compared the number and turnover of microaneurysms in 28 eyes that received three consecutive monthly faricimab injections with their fellow untreated eyes. They reported a significant reduction in the formation of new microaneurysms, a significant increase in turnover and significant decrease in new microaneurysm formation leading to a total reduction of 40% in microaneurysms in faricimab-treated eyes [186].

In summary, the pivotal registration trials YOSEMITE and RHINE have demonstrated the non-inferiority of intravitreal faricimab to intravitreal aflibercept in eyes with DME with respect to the change in BCVA from baseline to weeks 48, 52 and 56. These results were obtained with less injections than the eyes treated with faricimab. However, it must be remembered that in the aflibercept arms, eyes were treated according to the label and there was no possibility of extending the treatment beyond 8 weeks. The short-term results in routine clinical practice indicate that faricimab may rescue many eyes that were not responding well to aflibercept. In most of these eyes, the anatomy improved but the visual acuity did not. In contrast, treatment-naive eyes with DME had a good response to faricimab.

ii. Nesvacumab (REGN910-3) plus aflibercept

Nesvacumab (Regeneron, Tarrytown, NY, USA) is an IgG1 fully human monoclonal antibody that selectively inhibits Ang2 [189]. REGN910-3 is a co-formulation of a fixed dose of aflibercept (2 mg) and nesvacumab delivered as a single intravitreal injection [189]. There is a theoretical advantage of a fixed-dose combination such as nesvacumab plus aflibercept over a bispecific molecule like faricimab. A bispecific molecule cannot dose at different ratios limiting its flexibility. Furthermore, a bispecific molecule can only bind to a single ligand, either Ang2 or VEGF, per molecule [189]. Based on the results of the ONYX and RUBY trials, Regeneron decided to halt further development of nesvacumab [189,190].

The RUBY trial was a phase 2 study of 302 eyes that were followed for 36 weeks. Approximately 40% of patients had prior treatment for DME. This study compared the outcomes of eyes with DME following treatment with nesvacumab in two doses (3 mg and 6 mg) plus aflibercept to aflibercept monotherapy [189]. Neither dose of nesvacumab plus aflibercept provided an additional visual benefit over aflibercept monotherapy at 12 or 36 weeks failing to meet the primary endpoint of the study. However, patients treated with the high-dose combination exhibited improved anatomic outcomes. For instance, eyes treated with the combination of nesvacumab and aflibercept exhibited an increased reduction in CST, more eyes with foveal fluid resolution, more eyes with a normalization of macular thickness and a trend towards more patients improving in the DRSS over aflibercept monotherapy. Limitations of the study included its small sample size and its short duration. It is possible that this trial was underpowered to detect the effects of Ang2 inhibition [189].

### 4.2. VE-PTP Inhibitors

Tie2 activation can be restored by inhibiting VE-PTP activity in a ligand-independent manner [72,73].

- Razuprotafib

Razuprotafib (AKB-9778, Aerpio Therapeutics; Blue Ash, OH, USA) is a potent and selective inhibitor of the catalytic activity of VE-PTP [86]. Inhibition of VE-PTP activates the Tie2 signaling pathway and induces downstream signaling which improves vascular stability.

The TIME-1 was a small, dose escalation, phase 1B study of 24 patients with DME, where the subjects self-administered subcutaneous injections of 5 mg, 15 mg, 22.5 mg and 30 mg of razuprotafib twice daily for 4 weeks. It demonstrated that doses ≥ 15 mg reduced DME and improved vision in some patients. Both the anti-permeability effects and the visual outcomes were heterogeneous. In some patients, there was marked improvement, in others no changes and in others worsening. A modest, transient, dose-dependent reduction in systemic blood pressure was observed. This was most likely due to eNOS stimulation. Overall, it was felt that systemic administration of razuprotafib was well tolerated [191,192].

The TIME-2 study was a phase 2A randomized, placebo- and sham-injection controlled, double-masked study that was designed to assess the effects of subcutaneous razuprotafib 15 mg BID monotherapy plus sham intravitreal injections vs. razuprotafib 15 mg BID plus intravitreal ranibizumab 0.3 mg monthly vs. ranibizumab 0.3 mg monthly monotherapy plus subcutaneous placebo injection bid in 144 eyes with DME at 12 weeks. There was no improvement in the mean central subfield thickness (CST) in the razuprotafib monotherapy group. In contrast, both the ranibizumab and the ranibizumab plus razuprotafib groups experienced a mean decrease in CST. The combination arm had a larger reduction in CST than the ranibizumab monotherapy group. Similarly, the visual acuity outcomes again favored the ranibizumab and the ranibizumab plus razuprotafib groups over razuprotafib monotherapy. Again, the combination arm fared better than the ranibizumab monotherapy group. There was no difference between the three arms of the study with respect to a ≥2 step improvement in the diabetic retinopathy severity score (DRSS) [192,193].

The TIME-2b was a phase II clinical trial that assessed the safety and efficacy of razuprotafib in eyes with moderate to severe NPDR. Patients self-administered either 15 mg razuprotafib subcutaneously once daily or twice daily for 48 weeks. The primary endpoint of the trial, which was a ≥2 step improvement in the DRSS score at 48 weeks, was not met.

To the best of our knowledge, razuprotafib is no longer being developed for the treatment of DME or NPDR following the merger of Aerpio Therapeutics with Aadi Biosciences (https://www.globenewswire.com/news-release/2021/08/26/2287410/0/en/Aadi-Bioscience-Announces-Closing-of-Merger-with-Aerpio-Pharmaceuticals-and-155M-Private-Placement.html accessed on 12 August 2022).

ii. ARP-1536

VE-PTP is upregulated under hypoxic conditions in a mouse model of ischemic retinopathy [86]. A monoclonal antibody against the extracellular domain of VE-PTP delivered intravitreally, markedly reduced the area of retinal neovascularization. Similarly, in a laser induced CNV mouse model, intravitreal anti-VE-PTP reduced the amount of CNV [86]. Targeting of the extracellular domain of VE-PTP by monoclonal antibodies elicits endocytosis of the Tie2-bound VE-PTP molecules but not of the VE-cadherin-bound VE-PTP molecules, causing dissociation of VE-PTP from Tie2 which leads to Tie2 activation [73].

ARP-1536 (Aerpio Therapeutics; Blue Ash, OH, USA) is a humanized monoclonal antibody that is delivered intravitreally and inhibits VE-PTP by binding to its extracellular domain leading to an increase activation of Tie2 [194]. Similar to razuprotafib, to the best of our knowledge, it appears that ARP 1536 is no longer being developed.

### 4.3. Integrin Binding Peptide

Integrins are obligate heterodimeric transmembrane surface receptors consisting of an alpha and a beta subunit that are involved in cell–cell and cell–matrix adhesion and intracellular–extracellular and extracellular–intracellular signaling. So far, 18 alpha subunits and 8 beta subunits have been identified. Several combinations of these subunits constitute the 24 integrins. The integrin receptor family has been classified according to the ligand recognition pattern and their structural similarity into the following four groups: collagen-binding, laminin-binding, leukocyte-specific and RGD-binding integrins [195].

Integrins connect the ECM to cytoskeletal proteins and transduce biochemical signals across the plasma membrane to regulate cellular functions. The integrin and EC ectodomains couple the EC to the underlying ECM, whereas their intracellular domains recruit actin binding proteins. Integrins are connected to the actin cytoskeleton via focal adhesions through adapter and linker proteins. These include vinculin, alpha-actinin, talin and paxillin [196]. Cross talk between integrins and the Tie receptors coordinate several biological processes through common signaling pathways and downstream effectors including p85, FAK and Rho GTPases [197,198,199].

- Gersizangitide (AXT107)

Gersizangitide, formerly known as AXT107 (AsclepiX Therapeutics, Baltimore, MA, USA), is a biomimetic optimized non-RGD 20-mer ανβ3 and α5β3 integrin-binding peptide derived from collagen IV that acts as a Tie2 activator and VEGFR2 inhibitor [200].

Gersizangitide potentiates the activation of Tie2 by Ang2 by converting the endogeneously released Ang2 into a Tie2 agonist. It binds to α5β1 integrin and dissociates α5 and β1 integrins which leads to translocation and activation of Tie2 to the EC junctions. As a result, the F-actin and VE-cadherin are re-organized resulting in decreased permeability and leakage [201]. Confluent monolayers of microvascular endothelial cells were treated with gersizangitide and Ang2. Gersizangitide alone did not elicit phosphorylation of Tie2. The combination of gersizangitide and Ang2 led to a strong and robust phosphorylation of Tie2 followed by phosphorylation of Akt. In contrast, ERK1/2 was not affected [200,201,202].

In different animal models, gersizangitide suppressed type II and type III macular neovascularization, ischemia induced retinal neovascularization and VEGF-induced vascular leakage [203]. A combination of gersizangitide and aflibercept demonstrated synergism in the inhibition of subretinal neovascularization. Compared to aflibercept the reduction in vascular leakage lasted longer with gersizangitide [204]. After an intravitreal injection of gersizangitide, a gel-like depot forms outside of the visual axis which may act as a reservoir and may explain its longer-acting activity when compared to aflibercept. It did not elicit an increase in intraocular pressure, degradation of the ocular media in the visual axis and no retinal toxicity [200].

### 4.4. Surrobodies

A surrogate antibody or surrobody are naturally occurring precursors of antibodies that have high affinity to their antigen. They are based on the pre-B cell receptor (pre-BCR) which is transiently expressed during development of the antibody repertoire. Typically, antibodies are composed of identical pairs of light and heavy chains. In contrast, the pre-BCR consists of a heterohexameric complex of identical heavy chains. Each of these heavy chains is paired with a two-subunit surrogate light chain (SLC). Each of the two SLC components contains non-immunoglobulin-like peptide extensions. Surrobodies have several advantages over monoclonal antibodies. They are easier to produce and more stable. In addition, they can be designed to have a specific affinity for a particular antigen [205,206].

- RO101

Preclinical studies demonstrated that R010, a surrobody that binds both Ang2 and VEGF-A, decreased the total area of choroidal neovascularization (CNV) leakage in the laser-induced CNV rat model. The intravitreal half-life in rabbits was 6.75 days. R0101 inhibited human umbilical vein endothelial cell migration to the same extent as aflibercept. In vitro studies showed that R0101 bound to VEGF-A with a 3-fold higher affinity than faricimab. R0101 binding to Ang2 was 17-fold stronger than faricimab. Electrophysiologic studies did not reveal any retinal toxicities.

## 5. Conclusions

DR remains one of the leading causes of preventable blindness worldwide. Over the past three decades increasing levels of evidence have implicated the angiopoietins as playing key roles in vascular homeostasis. As a result, angiopoietins have emerged as an exciting therapeutic target for eyes with diabetic retinopathy and macular edema. Faricimab, the first bispecific antibody targeting Ang2 and VEGF-A, has recently been approved across the world. Its initial results are promising and may decrease the treatment burden and may improve outcomes in comparison to anti-VEGF monotherapy. Promising alternatives that also target the angiopoietin–Tie signaling pathway include bispecific surrobodies and integrin binding peptides.

## References

[1] Ogurtsova K., da Rocha Fernandes J.D., Huang Y., Linnenkamp U., Guariguata L., Cho N.H., Cavan D., Shaw J.E., Makaroff L.E. (2017). IDF Diabetes Atlas: Global estimates for the prevalence of diabetes for 2015 and 2040. Diabetes Res. Clin. Pract..

[2] Klein R., Klein B.E., Moss S.E. (1984). Visual impairment in diabetes. Ophthalmology.

[3] Williams R., Airey M., Baxter H., Forrester J., Kennedy-Martin T., Girach A. (2004). Epidemiology of diabetic retinopathy and macular oedema: A systematic review. Eye.

[4] Brown M.M., Brown G.C., Sharma S., Shah G. (1999). Utility values and diabetic retinopathy. Am. J. Ophthalmol..

[5] Javitt J.C., Aiello L.P., Chiang Y., Ferris F.L., Canner J.K., Greenfield S. (1994). Preventive eye care in people with diabetes is cost-saving to the federal government. Implications for health-care reform. Diabetes Care.

[6] Javitt J.C., Aiello L.P. (1996). Cost-effectiveness of detecting and treating diabetic retinopathy. Ann. Intern. Med..

[7] Tan T.E., Wong T.Y. (2023). Diabetic retinopathy: Looking forward to 2030. Front. Endocrinol.

[8] Engerman R.L., Kern T.S. (1995). Retinopathy in animal models of diabetes. Diabetes/Metabolism Res. Rev..

[9] Klein R., Klein B.E., Moss S.E., Cruickshanks K.J. (1994). The Wisconsin Epidemiologic Study of diabetic retinopathy, X.I.V. Ten-year incidence and progression of diabetic retinopathy. Arch. Ophthalmol..

[10] Singh A., Stewart J.M. (2009). Pathophysiology of diabetic macular edema. Int. Ophthalmol. Clin..

[11] Wu L., Acon D., Wu A., Wu M. (2019). Vascular endothelial growth factor inhibition and proliferative diabetic retinopathy, a changing treatment paradigm?. Taiwan J. Ophthalmol..

[12] Grant M.B., Afzal A., Spoerri P., Pan H., Shaw L.C., Mames R.N. (2004). The role of growth factors in the pathogenesis of diabetic retinopathy. Expert Opin. Investig. Drugs.

[13] Owen L.A., Hartnett M.E. (2013). Soluble mediators of diabetic macular edema: The diagnostic role of aqueous VEGF and cytokine levels in diabetic macular edema. Curr. Diabetes Rep..

[14] Brown D.M., Schmidt-Erfurth U., Do D.V., Holz F.G., Boyer D.S., Midena E., Heier J.S., Terasaki H., Kaiser P.K., Marcus D.M. (2015). Intravitreal Aflibercept for Diabetic Macular Edema: 100-Week Results From the VISTA and VIVID Studies. Ophthalmology.

[15] Nguyen Q.D., Brown D.M., Marcus D.M., Boyer D.S., Patel S., Feiner L., Gibson A., Sy J., Rundle A.C., Hopkins J.J. (2012). Ranibizumab for diabetic macular edema: Results from 2 phase III randomized trials: RISE and RIDE. Ophthalmology.

[16] Rajendram R., Fraser-Bell S., Kaines A., Michaelides M., Hamilton R.D., Esposti S.D., Peto T., Egan C., Bunce C., Leslie R.D. (2012). A 2-year prospective randomized controlled trial of intravitreal bevacizumab or laser therapy (BOLT) in the management of diabetic macular edema: 24-month data: Report 3. Arch. Ophthalmol..

[17] Arevalo J.F., Lasave A.F., Wu L., Acon D., Farah M.E., Gallego-Pinazo R., Alezzandrini A.A., Fortuna V., Quiroz-Mercado H., Salcedo-Villanueva G. (2016). Intravitreal bevacizumab for diabetic macular oedema: 5-year results of the Pan-American Collaborative Retina Study group. Br. J. Ophthalmol..

[18] Ciulla T.A., Bracha P., Pollack J., Williams D.F. (2018). Real-world Outcomes of Anti-Vascular Endothelial Growth Factor Therapy in Diabetic Macular Edema in the United States. Ophthalmol. Retin..

[19] Wells J.A., Glassman A.R., Ayala A.R., Jampol L.M., Bressler N.M., Bressler S.B., Brucker A.J., Ferris F.L., Hampton G.R., Jhaveri C. (2016). Aflibercept, Bevacizumab, or Ranibizumab for Diabetic Macular Edema: Two-Year Results from a Comparative Effectiveness Randomized Clinical Trial. Ophthalmology.

[20] Davis S., Aldrich T.H., Jones P.F., Acheson A., Compton D.L., Jain V., Ryan T.E., Bruno J., Radziejewski C., Maisonpierre P.C. (1996). Isolation of angiopoietin-1, a ligand for the TIE2 receptor, by secretion-trap expression cloning. Cell.

[21] Maisonpierre P.C., Goldfarb M., Yancopoulos G.D., Gao G. (1993). Distinct rat genes with related profiles of expression define a TIE receptor tyrosine kinase family. Oncogene.

[22] Maisonpierre P.C., Suri C., Jones P.F., Bartunkova S., Wiegand S.J., Radziejewski C., Compton D., McClain J., Aldrich T.H., Papadopoulos N. (1997). Angiopoietin-2, a natural antagonist for Tie2 that disrupts in vivo angiogenesis. Science.

[23] Park S.W., Yun J.H., Kim J.H., Kim K.W., Cho C.H., Kim J.H. (2014). Angiopoietin 2 induces pericyte apoptosis via alpha3beta1 integrin signaling in diabetic retinopathy. Diabetes.

[24] Yun J.H., Park S.W., Kim J.H., Park Y.J., Cho C.H., Kim J.H. (2016). Angiopoietin 2 induces astrocyte apoptosis via alphavbeta5-integrin signaling in diabetic retinopathy. Cell Death. Dis..

[25] Zhou H., Chen T., Li Y., You J., Deng X., Chen N., Li T., Zheng Y., Li R., Luo M. (2022). Glycation of Tie-2 Inhibits Angiopoietin-1 Signaling Activation and Angiopoietin-1-Induced Angiogenesis. Int. J. Mol. Sci..

[26] Joussen A.M., Poulaki V., Tsujikawa A., Qin W., Qaum T., Xu Q., Moromizato Y., Bursell S.E., Wiegand S.J., Rudge J. (2002). Suppression of diabetic retinopathy with angiopoietin-1. Am. J. Pathol..

[27] Peters S., Cree I.A., Alexander R., Turowski P., Ockrim Z., Patel J., Boyd S.R., Joussen A.M., Ziemssen F., Hykin P.G. (2007). Angiopoietin modulation of vascular endothelial growth factor: Effects on retinal endothelial cell permeability. Cytokine.

[28] Rangasamy S., Srinivasan R., Maestas J., McGuire P.G., Das A. (2011). A potential role for angiopoietin 2 in the regulation of the blood-retinal barrier in diabetic retinopathy. Investig. Opthalmology Vis. Sci..

[29] Boone C.V., Wykoff C.C., Fine H.F. (2022). From Bench to Bedside: Faricimab Enters the Clinic. Ophthalmic Surg. Lasers Imaging Retin..

[30] Mustonen T., Alitalo K. (1995). Endothelial receptor tyrosine kinases involved in angiogenesis. J. Cell Biol..

[31] Davis S., Papadopoulos N., Aldrich T.H., Maisonpierre P.C., Huang T., Kovac L., Xu A., Leidich R., Radziejewska E., Rafique A. (2002). Angiopoietins have distinct modular domains essential for receptor binding, dimerization and superclustering. Nat. Struct. Biol..

[32] Procopio W.N., Pelavin P.I., Lee W.M., Yeilding N.M. (1999). Angiopoietin-1 and -2 coiled coil domains mediate distinct homo-oligomerization patterns, but fibrinogen-like domains mediate ligand activity. J. Biol. Chem..

[33] Yee V.C., Pratt K.P., Cote H.C., Trong I.L., Chung D.W., Davie E.W., Stenkamp R.E., Teller D.C. (1997). Crystal structure of a 30 kDa C-terminal fragment from the gamma chain of human fibrinogen. Structure.

[34] Zhou B.O., Ding L., Morrison S.J. (2015). Hematopoietic stem and progenitor cells regulate the regeneration of their niche by secreting Angiopoietin-1. eLife.

[35] Park D.Y., Lee J., Kim J., Kim K., Hong S., Han S., Kubota Y., Augustin H.G., Ding L., Kim J.W. (2017). Plastic roles of pericytes in the blood-retinal barrier. Nat. Commun..

[36] Tripathi B.J., Tripathi R.C. (1989). Neural crest origin of human trabecular meshwork and its implications for the pathogenesis of glaucoma. Am. J. Ophthalmol..

[37] Gage P.J., Rhoades W., Prucka S.K., Hjalt T. (2005). Fate maps of neural crest and mesoderm in the mammalian eye. Investig. Ophthalmol. Vis. Sci..

[38] Bharti K., Nguyen M.T., Skuntz S., Bertuzzi S., Arnheiter H. (2006). The other pigment cell: Specification and development of the pigmented epithelium of the vertebrate eye. Pigment. Cell Res..

[39] Kim K.T., Choi H.H., Steinmetz M.O., Maco B., Kammerer R.A., Ahn S.Y., Kim H.Z., Lee G.M., Koh G.Y. (2005). Oligomerization and multimerization are critical for angiopoietin-1 to bind and phosphorylate Tie2. J. Biol. Chem..

[40] Saharinen P., Kerkela K., Ekman N., Marron M., Brindle N., Lee G.M., Augustin H., Koh G.Y., Alitalo K. (2005). Multiple angiopoietin recombinant proteins activate the Tie1 receptor tyrosine kinase and promote its interaction with Tie2. J. Cell Biol..

[41] Dallabrida S.M., Ismail N., Oberle J.R., Himes B.E., Rupnick M.A. (2005). Angiopoietin-1 promotes cardiac and skeletal myocyte survival through integrins. Circ. Res..

[42] Carlson T.R., Feng Y., Maisonpierre P.C., Mrksich M., Morla A.O. (2001). Direct cell adhesion to the angiopoietins mediated by integrins. J. Biol. Chem..

[43] Huang Y.Q., Li J.J., Hu L., Karpatkin S. (2002). Thrombin induces increased expression and secretion of angiopoietin-2 from human umbilical vein endothelial cells. Blood.

[44] Kelly B.D., Hackett S.F., Hirota K., Oshima Y., Cai Z., Berg-Dixon S., Rowan A., Yan Z., Campochiaro P.A., Semenza G.L. (2003). Cell type-specific regulation of angiogenic growth factor gene expression and induction of angiogenesis in nonischemic tissue by a constitutively active form of hypoxia-inducible factor 1. Circ. Res..

[45] Wang Q., Lash G.E. (2017). Angiopoietin 2 in placentation and tumor biology: The yin and yang of vascular biology. Placenta.

[46] Sfiligoi C., de Luca A., Cascone I., Sorbello V., Fuso L., Ponzone R., Biglia N., Audero E., Arisio R., Bussolino F. (2002). Angiopoietin-2 expression in breast cancer correlates with lymph node invasion and short survival. Int. J. Cancer.

[47] Hu B., Cheng S.Y. (2009). Angiopoietin-2: Development of inhibitors for cancer therapy. Curr. Oncol. Rep..

[48] Hammes H.P., Lin J., Wagner P., Feng Y., Vom Hagen F., Krzizok T., Renner O., Breier G., Brownlee M., Deutsch U. (2004). Angiopoietin-2 causes pericyte dropout in the normal retina: Evidence for involvement in diabetic retinopathy. Diabetes.

[49] Fiedler U., Scharpfenecker M., Koidl S., Hegen A., Grunow V., Schmidt J.M., Kriz W., Thurston G., Augustin H.G. (2004). The Tie-2 ligand angiopoietin-2 is stored in and rapidly released upon stimulation from endothelial cell Weibel-Palade bodies. Blood.

[50] Saharinen P., Eklund L., Alitalo K. (2017). Therapeutic targeting of the angiopoietin-TIE pathway. Nat. Rev. Drug Discov..

[51] Valenzuela D.M., Griffiths J.A., Rojas J., Aldrich T.H., Jones P.F., Zhou H., McClain J., Copeland N.G., Gilbert D.J., Jenkins N.A. (1999). Angiopoietins 3 and 4: Diverging gene counterparts in mice and humans. Proc. Natl. Acad. Sci. USA.

[52] Elamaa H., Kihlstrom M., Kapiainen E., Kaakinen M., Miinalainen I., Ragauskas S., Cerrada-Gimenez M., Mering S., Nätynki M., Eklund L. (2018). Angiopoietin-4-dependent venous maturation and fluid drainage in the peripheral retina. eLife.

[53] Kapiainen E., Elamaa H., Miinalainen I., Izzi V., Eklund L. (2022). Cooperation of Angiopoietin-2 and Angiopoietin-4 in Schlemm’s Canal Maintenance. Investig. Ophthalmol. Vis. Sci..

[54] Ullrich A., Schlessinger J. (1990). Signal transduction by receptors with tyrosine kinase activity. Cell.

[55] Schlessinger J., Ullrich A. (1992). Growth factor signaling by receptor tyrosine kinases. Neuron.

[56] Dumont D.J., Yamaguchi T.P., Conlon R.A., Rossant J., Breitman M.L. (1992). Tek, a novel tyrosine kinase gene located on mouse chromosome 4, is expressed in endothelial cells and their presumptive precursors. Oncogene.

[57] Runting A.S., Stacker S.A., Wilks A.F. (1993). tie2, a putative protein tyrosine kinase from a new class of cell surface receptor. Growth Factors.

[58] Barton W.A., Dalton A.C., Seegar T.C., Himanen J.P., Nikolov D.B. (2014). Tie2 and Eph receptor tyrosine kinase activation and signaling. Cold Spring Harb. Perspect. Biol..

[59] Yu X., Seegar T.C., Dalton A.C., Tzvetkova-Robev D., Goldgur Y.R., Tajashankar K.R., Nikolov D.B., Barton W.A. (2013). Structural basis for angiopoietin-1-mediated signaling initiation. Proc. Natl. Acad. Sci. USA.

[60] Dumont D.J., Fong G.H., Puri M.C., Gradwohl G., Alitalo K., Breitman M.L. (1995). Vascularization of the mouse embryo: A study of flk-1, tek, tie, and vascular endothelial growth factor expression during development. Dev. Dyn..

[61] Sato T.N., Qin Y., Kozak C.A., Audus K.L. (1993). Tie-1 and tie-2 define another class of putative receptor tyrosine kinase genes expressed in early embryonic vascular system. Proc. Natl. Acad. Sci. USA.

[62] Ziegler S.F., Bird T.A., Schneringer J.A., Schooley K.A., Baum P.R. (1993). Molecular cloning and characterization of a novel receptor protein tyrosine kinase from human placenta. Oncogene.

[63] Schnurch H., Risau W. (1993). Expression of tie-2, a member of a novel family of receptor tyrosine kinases, in the endothelial cell lineage. Development.

[64] Macdonald P.R., Progias P., Ciani B., Patel S., Mayer U., Steinmetz M.O., Kammerer R.A. (2006). Structure of the extracellular domain of Tie receptor tyrosine kinases and localization of the angiopoietin-binding epitope. J. Biol. Chem..

[65] Partanen J., Armstrong E., Makela T.P., Korhonen J., Sandberg M., Renkonen R.S., Huebner K., Alitalo K. (1992). A novel endothelial cell surface receptor tyrosine kinase with extracellular epidermal growth factor homology domains. Mol. Cell Biol..

[66] Kim K.L., Shin I.S., Kim J.M., Choi J.H., Byun J., Jeon E.S., Suh W., Kim D.K. (2006). Interaction between Tie receptors modulates angiogenic activity of angiopoietin2 in endothelial progenitor cells. Cardiovasc. Res..

[67] Seegar T.C., Eller B., Tzvetkova-Robev D., Kolev M.V., Hemderson S.C., Nikolov D.B., Barton W.A. (2010). Tie1-Tie2 interactions mediate functional differences between angiopoietin ligands. Mol. Cell.

[68] Wong A.L., Haroon Z.A., Werner S., Dewhirst M.W., Greenberg C.S., Peters K.G. (1997). Tie2 expression and phosphorylation in angiogenic and quiescent adult tissues. Circ. Res..

[69] Krueger N.X., Streuli M., Saito H. (1990). Structural diversity and evolution of human receptor-like protein tyrosine phosphatases. EMBO J..

[70] Braun L.J., Zinnhardt M., Vockel M., Drexel H.C., Peters K., Vestweber D. (2019). VE-PTP inhibition stabilizes endothelial junctions by activating FGD5. Embo Rep..

[71] Mellberg S., Dimberg A., Bahram F., Makoto H., Rennel E., Ameur A., Westholm J.O., Larsson E., Lindahl P., Cross M.J. (2009). Transcriptional profiling reveals a critical role for tyrosine phosphatase VE-PTP in regulation of VEGFR2 activity and endothelial cell morphogenesis. FASEB J..

[72] Yacyshyn O.K., Lai P.F., Forse K., Teichert-Kuliszewska K., Jurasz P., Stewart D.J. (2009). Tyrosine phosphatase beta regulates angiopoietin-Tie2 signaling in human endothelial cells. Angiogenesis.

[73] Winderlich M., Keller L., Cagna G., Broermann A., Kamenyeva O., Kiefer F., Deutsch U., Nottebaum A.F., Vestweber D. (2009). VE-PTP controls blood vessel development by balancing Tie-2 activity. J. Cell Biol..

[74] Fachinger G., Deutsch U., Risau W. (1999). Functional interaction of vascular endothelial-protein-tyrosine phosphatase with the angiopoietin receptor Tie-2. Oncogene.

[75] Vestweber D. (2021). Vascular Endothelial Protein Tyrosine Phosphatase Regulates Endothelial Function. Physiology.

[76] Allingham M.J., van Buul J.D., Burridge K. (2007). ICAM-1-mediated, Src- and Pyk2-dependent vascular endothelial cadherin tyrosine phosphorylation is required for leukocyte transendothelial migration. J. Immunol..

[77] Turowski P., Martinelli R., Crawford R., Wateridge D., Papageorgiou A.P., Lampugnani M.G., Gamp A.C., Vestweber D., Adamson P., Dejana E. (2008). Phosphorylation of vascular endothelial cadherin controls lymphocyte emigration. J. Cell Sci..

[78] Wallez Y., Cand F., Cruzalegui F., Souchelnytskyi S., Vilgrain I., Huber P. (2006). Src kinase phosphorylates vascular endothelial-cadherin in response to vascular endothelial growth factor: Identification of tyrosine 685 as the unique target site. Oncogene.

[79] Wessel F., Winderlich M., Holm M., Frye M., Rivera-Galdos R., Vockel M., Linnepe R., Ipe U., Stadtmann A., Zarbock A. (2014). Leukocyte extravasation and vascular permeability are each controlled in vivo by different tyrosine residues of VE-cadherin. Nat. Immunol..

[80] Orsenigo F., Giampietro C., Ferrari A., Corada M., Galaup A., Sigismund S., Ristagno G., Maddaluno L., Koh G.Y., Franco D. (2012). Phosphorylation of VE-cadherin is modulated by haemodynamic forces and contributes to the regulation of vascular permeability in vivo. Nat. Commun..

[81] Corada M., Mariotti M., Thurston G., Smith K., Kunkel R., Brockhaus M., Lampugnani M.G., Martin-Padura I., Stoppacciaro A., Ruco L. (1999). Vascular endothelial-cadherin is an important determinant of microvascular integrity in vivo. Proc. Natl. Acad. Sci. USA.

[82] Gotsch U., Borges E., Bosse R., Böggemeyer E., Simon M., Mossmann H., Vestweber D. (1997). VE-cadherin antibody accelerates neutrophil recruitment in vivo. J. Cell Sci..

[83] Breviario F., Caveda L., Corada M., Martin-Padura I., Navarro P., Golay J., Introna M., Gulino D., Lampugnani M.G., Dejana E. (1995). Functional properties of human vascular endothelial cadherin (7B4/cadherin-5), an endothelium-specific cadherin. Arterioscler. Thromb. Vasc. Biol..

[84] Nawroth R., Poell G., Ranft A., Kloep S., Samulowitz U., Fachinger G., Golding M., Shima D.T., Deutsch U., Vestweber D. (2002). VE-PTP and VE-cadherin ectodomains interact to facilitate regulation of phosphorylation and cell contacts. EMBO J..

[85] Nottebaum A.F., Cagna G., Winderlich M., Gamp A.C., Linnepe R., Polaschegg C., Filippova K., Lyck R., Engelhardt B., Kamenyeva O. (2008). VE-PTP maintains the endothelial barrier via plakoglobin becomes dissociated from VE-cadherin by leukocytes by, V.E.G.F. J. Exp. Med..

[86] Shen J., Frye M., Lee B.L., Reinardy J.L., McClung J.M., Ding K., Kojima M., Xia H., Seidel C., Lima e Silva R. (2014). Targeting VE-PTP activates TIE2 and stabilizes the ocular vasculature. J. Clin. Investig..

[87] Dominguez M.G., Hughes V.C., Pan L., Simmons M., Daly C., Anderson K., Noguera-Troise I., Murphy A.J., Valenzuela D.M., Davis S. (2007). Vascular endothelial tyrosine phosphatase (VE-PTP)-null mice undergo vasculogenesis but die embryonically because of defects in angiogenesis. Proc. Natl. Acad. Sci. USA.

[88] Frye M., Dierkes M., Kuppers V., Vockel M., Tomm J., Zeuschner D., Rossaint J., Zarbock A., Koh G.Y., Peters K. (2015). Interfering with VE-PTP stabilizes endothelial junctions in vivo via Tie-2 in the absence of VE-cadherin. J. Exp. Med..

[89] Baumer S., Keller L., Holtmann A., Funke R., August B., Gamp A., Wolburg H., Wolburg-Buchholz K., Deutsch U., Vestweber D. (2006). Vascular endothelial cell-specific phosphotyrosine phosphatase (VE-PTP) activity is required for blood vessel development. Blood.

[90] Dumont D.J., Gradwohl G., Fong G.H., Puri M.C., Gertsenstein M., Auerbach A., Breitman M.L. (1994). Dominant-negative and targeted null mutations in the endothelial receptor tyrosine kinase, tek, reveal a critical role in vasculogenesis of the embryo. Genes Dev..

[91] Suri C., Jones P.F., Patan S., Bartunkova S., Maisonpierre P.C., Davis S., Sato T.N., Yancopoulos G.D. (1996). Requisite role of angiopoietin-1, a ligand for the TIE2 receptor, during embryonic angiogenesis. Cell.

[92] Reynolds L.P., Killilea S.D., Redmer D.A. (1992). Angiogenesis in the female reproductive system. FASEB J..

[93] Ohashi H., Takagi H., Koyama S., Oh H., Watanabe D., Antonetti D.A., Matsubara T., Nagai K., Arai H., Kita T. (2004). Alterations in expression of angiopoietins and the Tie-2 receptor in the retina of streptozotocin induced diabetic rats. Mol. Vis..

[94] Heier J.S., Singh R.P., Wykoff C.C., Csaky K.G., Lai T.Y.Y., Loewenstein A., Schlottmann P.G., Paris L.P., Westenskow P.D., Quezada-Ruiz C. (2021). The Angiopoietin/Tie Pathway in Retinal Vascular Diseases: A Review. Retina.

[95] Hammes H.P., Lin J., Renner O., Wang Y., Stock O., Vom Hagen F., Wolburg H., Hoffmann S., Deutsch U., Hammes H.P. (2002). Pericytes and the pathogenesis of diabetic retinopathy. Diabetes.

[96] Tao-Cheng J.H., Nagy Z., Brightman M.W. (1987). Tight junctions of brain endothelium in vitro are enhanced by astroglia. J. Neurosci..

[97] Janzer R.C., Raff M.C. (1987). Astrocytes induce blood-brain barrier properties in endothelial cells. Nature.

[98] Gardner T.W., Lieth E., Khin S.A., Barber A.J., Bonsall D.J., Lesher T., Rice K., Brennan W.A. (1997). Astrocytes increase barrier properties and ZO-1 expression in retinal vascular endothelial cells. Investig. Ophthalmol. Vis. Sci..

[99] Gaonac’h-Lovejoy V., Boscher C., Delisle C., Gratton J.P. (2020). Rap1 is Involved in Angiopoietin-1-Induced Cell-Cell Junction Stabilization and Endothelial Cell Sprouting. Cells.

[100] Peters K.G., Kontos C.D., Lin P.C., Wong A.L., Rao P., Huang L., Dewhirst M.W., Sankar S. (2004). Functional significance of Tie2 signaling in the adult vasculature. Recent Prog. Horm. Res..

[101] Asahara T., Chen D., Takahashi T., Fujikawa K., Kearney M., Magner M., Yancopoulos G.D., Isner J.M. (1998). Tie2 receptor ligands, angiopoietin-1 and angiopoietin-2, modulate VEGF-induced postnatal neovascularization. Circ. Res..

[102] Lin P., Polverini P., Dewhirst M., Shan S., Rao P.S., Peters K. (1997). Inhibition of tumor angiogenesis using a soluble receptor establishes a role for Tie2 in pathologic vascular growth. J. Clin. Investig..

[103] Eklund L., Olsen B.R. (2006). Tie receptors and their angiopoietin ligands are context-dependent regulators of vascular remodeling. Exp. Cell Res..

[104] Fukuhara S., Sako K., Minami T., Noda K., Kim H.Z., Kodama T., Shibuya M., Takakura N., Koh G.Y., Mochizuki N. (2008). Differential function of Tie2 at cell-cell contacts and cell-substratum contacts regulated by angiopoietin-1. Nat. Cell Biol..

[105] Saharinen P., Eklund L., Miettinen J., Wirkkala R., Anisimov A., Winderlich M., Nottebaum A., Vestweber D., Deutsch U., Koh G.Y. (2008). Angiopoietins assemble distinct Tie2 signalling complexes in endothelial cell-cell and cell-matrix contacts. Nat. Cell Biol..

[106] Thurston G., Daly C. (2012). The complex role of angiopoietin-2 in the angiopoietin-tie signaling pathway. Cold Spring Harb. Perspect. Med..

[107] Harfouche R., Hussain S.N. (2006). Signaling and regulation of endothelial cell survival by angiopoietin-2. Am. J. Physiol. Heart Circ. Physiol..

[108] DeBusk L.M., Hallahan D.E., Lin P.C. (2004). Akt is a major angiogenic mediator downstream of the Ang1/Tie2 signaling pathway. Exp. Cell Res..

[109] Hackett S.F., Ozaki H., Strauss R.W., Wahlin K., Suri C., Maisonpierre P., Yancopoulos G., Campochiaro P.A. (2000). Angiopoietin 2 expression in the retina: Upregulation during physiologic and pathologic neovascularization. J. Cell. Physiol..

[110] Gale N.W., Thurston G., Hackett S.F., Renard R., Wang Q., McClain J., Martin C., Witte C., Witte M.H., Jackson D. (2002). Angiopoietin-2 is required for postnatal angiogenesis and lymphatic patterning, and only the latter role is rescued by Angiopoietin-1. Dev. Cell.

[111] Oh H., Takagi H., Suzuma K., Otani A., Matsumura M., Honda Y. (1999). Hypoxia and vascular endothelial growth factor selectively up-regulate angiopoietin-2 in bovine microvascular endothelial cells. J. Biol. Chem..

[112] Hackett S.F., Wiegand S., Yancopoulos G., Campochiaro P.A. (2002). Angiopoietin-2 plays an important role in retinal angiogenesis. J. Cell. Physiol..

[113] Park Y.S., Kim N.H., Jo I. (2003). Hypoxia and vascular endothelial growth factor acutely up-regulate angiopoietin-1 and Tie2 mRNA in bovine retinal pericytes. Microvasc. Res..

[114] Mandriota S.J., Pepper M.S. (1998). Regulation of angiopoietin-2 mRNA levels in bovine microvascular endothelial cells by cytokines and hypoxia. Circ. Res..

[115] Wagner D.D. (1993). The Weibel-Palade body: The storage granule for von Willebrand factor and P-selectin. Thromb. Haemost..

[116] Hop C., Guilliatt A., Daly M., de Leeuw H.P., Brinkman H.J., Peake I.R., van Mourik J.A., Pannekoek H. (2000). Assembly of multimeric von Willebrand factor directs sorting of P-selectin. Arter. Thromb. Vasc. Biol..

[117] Teichert-Kuliszewska K., Maisonpierre P.C., Jones N., Campbell A.I., Master Z., Bendeck M.P., Alitalo K., Dumont D.J., Yancopoulos G.D., Stewart D.J. (2001). Biological action of angiopoietin-2 in a fibrin matrix model of angiogenesis is associated with activation of Tie2. Cardiovasc. Res..

[118] Augustin H.G., Koh G.Y., Thurston G., Alitalo K. (2009). Control of vascular morphogenesis and homeostasis through the angiopoietin-Tie system. Nat. Rev. Mol. Cell Biol..

[119] Mochizuki Y., Nakamura T., Kanetake H., Kanda S. (2002). Angiopoietin 2 stimulates migration and tube-like structure formation of murine brain capillary endothelial cells through c-Fes and c-Fyn. J. Cell Sci..

[120] Zhang Y., Kontos C.D., Annex B.H., Popel A.S. (2021). A systems biology model of junctional localization and downstream signaling of the Ang–Tie signaling pathway. Npj Syst. Biol. Appl..

[121] Cai J., Kehoe O., Smith G.M., Hykin P., Boulton M.E. (2008). The angiopoietin/Tie-2 system regulates pericyte survival and recruitment in diabetic retinopathy. Investig. Ophthalmol. Vis. Sci..

[122] Yao D., Taguchi T., Matsumura T., Pestell R., Edelstein D., Giardino I., Suske G., Rabbani N., Thornalley P.J., Sarthy V.P. (2007). High glucose increases angiopoietin-2 transcription in microvascular endothelial cells through methylglyoxal modification of, m.S.i.n.3.A. J. Biol. Chem..

[123] Okamoto T., Yamagishi S., Inagaki Y., Amano S., Koga K., Abe R., Takeuchi M., Ohno S., Yoshimura A., Makita Z. (2002). Angiogenesis induced by advanced glycation end products and its prevention by cerivastatin. FASEB J..

[124] Brownlee M. (2001). Biochemistry and molecular cell biology of diabetic complications. Nature.

[125] Gamble J.R., Drew J., Trezise L., Underwood A., Parsons M., Kasminkas L., Rudge J., Yancopoulos G., Vadas M.A. (2000). Angiopoietin-1 is an antipermeability and anti-inflammatory agent in vitro and targets cell junctions. Circ. Res..

[126] Gavard J., Patel V., Gutkind J.S. (2008). Angiopoietin-1 prevents VEGF-induced endothelial permeability by sequestering Src through mDia. Dev. Cell.

[127] Hwang J.A., Lee E.H., Lee S.D., Park J.B., Jeon B.H., Cho C.H. (2009). COMP-Ang1 ameliorates leukocyte adhesion and reinforces endothelial tight junctions during endotoxemia. Biochem. Biophys. Res. Commun..

[128] Miyamoto K., Khosrof S., Bursell S.E., Rohan R., Murata T., Clermont A.C., Aiello L.P., Ogura Y., Adamis A.P. (1999). Prevention of leukostasis and vascular leakage in streptozotocin-induced diabetic retinopathy via intercellular adhesion molecule-1 inhibition. Proc. Natl. Acad. Sci. USA.

[129] Joussen A.M., Murata T., Tsujikawa A., Kirchhof B., Bursell S.E., Adamis A.P. (2001). Leukocyte-mediated endothelial cell injury and death in the diabetic retina. Am. J. Pathol..

[130] Feng Y., Gross S., Wolf N.M., Butenschon V.M., Qiu Y., Devraj K., Liebner S., Kroll J., Skolnik E.Y., Hammes H.P. (2014). Nucleoside diphosphate kinase B regulates angiogenesis through modulation of vascular endothelial growth factor receptor type 2 and endothelial adherens junction proteins. Arter. Thromb. Vasc. Biol..

[131] Gross S., Devraj K., Feng Y., Macas J., Liebner S., Wieland T. (2016). Nucleoside diphosphate kinase B regulates angiogenic responses in the endothelium via caveolae formation and c-Src-mediated caveolin-1 phosphorylation. J. Cereb. Blood Flow Metab..

[132] Qiu Y., Zhao D., Butenschon V.M., Bauer A.T., Schneider S.W., Skolnik E.Y., Hammes H.P., Wieland T., Feng Y. (2015). Nucleoside diphosphate kinase B deficiency causes a diabetes-like vascular pathology via up-regulation of endothelial angiopoietin-2 in the retina. Acta Diabetol..

[133] Danser A.H., Derkx F.H., Admiraal P.J., Deinum J., de Jong P.T., Schalekamp M.A. (1994). Angiotensin levels in the eye. Investig. Ophthalmol. Vis. Sci..

[134] Danser A.H., van den Dorpel M.A., Deinum J., Derkx F.H., Franken A.A., Peperkamp E., de Jong P.T., Schalekamp M.A. (1989). Renin, prorenin, and immunoreactive renin in vitreous fluid from eyes with and without diabetic retinopathy. J. Clin. Endocrinol. Metab..

[135] Otani A., Takagi H., Oh H., Suzuma K., Matsumura M., Ikeda E., Honda Y. (2000). Angiotensin II-stimulated vascular endothelial growth factor expression in bovine retinal pericytes. Investig. Ophthalmol. Vis. Sci..

[136] Otani A., Takagi H., Oh H., Koyama S., Honda Y. (2001). Angiotensin II induces expression of the Tie2 receptor ligand, angiopoietin-2, in bovine retinal endothelial cells. Diabetes.

[137] Funatsu H., Yamashita H., Ikeda T., Mimura T., Eguchi S., Hori S. (2003). Vitreous levels of interleukin-6 and vascular endothelial growth factor are related to diabetic macular edema. Ophthalmology.

[138] Yu H., Pardoll D., Jove R. (2009). STATs in cancer inflammation and immunity: A leading role for STAT3. Nat. Rev. Cancer.

[139] Yun J.H., Park S.W., Kim K.J., Bae J.S., Lee E.H., Paek S.H., Kim S.U., Ye S., Kim J.H., Cho C.H. (2017). Endothelial STAT3 Activation Increases Vascular Leakage Through Downregulating Tight Junction Proteins: Implications for Diabetic Retinopathy. J. Cell. Physiol..

[140] Yun J.H., Han M.H., Jeong H.S., Lee D.H., Cho C.H. (2019). Angiopoietin 1 attenuates interleukin-6-induced endothelial cell permeability through SHP-1. Biochem. Biophys. Res. Commun..

[141] Feng Y., vom Hagen F., Pfister F., Djokic S., Hoffmann S., Back W., Wagner P., Lin J., Deutsch U., Hammes H.P. (2007). Impaired pericyte recruitment and abnormal retinal angiogenesis as a result of angiopoietin-2 overexpression. Arthritis Res. Ther..

[142] Bresnick G.H., Engerman R., Davis M.D., de Venecia G., Myers F.L. (1976). Patterns of ischemia in diabetic retinopathy. Trans. Sect. Ophthalmol. Am. Acad. Ophthalmol. Otolaryngol..

[143] Kohner E.M., Henkind P. (1970). Correlation of fluorescein angiogram and retinal digest in diabetic retinopathy. Am. J. Ophthalmol..

[144] Zhang Y., Stone J. (1997). Role of astrocytes in the control of developing retinal vessels. Investig. Ophthalmol. Vis. Sci..

[145] Pfister F., Wang Y., Schreiter K., vom Hagen F., Altvater K., Hoffmann S., Deutsch U., Hammes H.P., Feng Y. (2009). Retinal overexpression of angiopoietin-2 mimics diabetic retinopathy and enhances vascular damages in hyperglycemia. Acta Diabetol..

[146] Pfister F., Feng Y., vom Hagen F., Hoffmann S., Molema G., Hillebrands J.L., Shani M., Deutsch U., Hammes H.P. (2008). Pericyte migration: A novel mechanism of pericyte loss in experimental diabetic retinopathy. Diabetes.

[147] Feng Y., Pfister F., Schreiter K., Wang Y., Stock O., Vom Hagen F., Wolburg H., Hoffmann S., Deutsch U., Hammes H.P. (2008). Angiopoietin-2 deficiency decelerates age-dependent vascular changes in the mouse retina. Cell. Physiol. Biochem..

[148] Feng Y., Vom Hagen F., Wang Y., Beck S., Schreiter K., Pfister F., Hoffmann S., Wagner P., Seeliger M., Molema G. (2009). The absence of angiopoietin-2 leads to abnormal vascular maturation and persistent proliferative retinopathy. Thromb. Haemost..

[149] Lobov I.B., Brooks P.C., Lang R.A. (2002). Angiopoietin-2 displays VEGF-dependent modulation of capillary structure and endothelial cell survival in vivo. Proc. Natl. Acad. Sci. USA.

[150] Jain R.K. (2003). Molecular regulation of vessel maturation. Nat. Med..

[151] Hanahan D. (1997). Signaling vascular morphogenesis and maintenance. Science.

[152] Carmeliet P. (2003). Angiogenesis in health and disease. Nat. Med..

[153] Kim H., Koh G.Y. (2011). Ang2, the instigator of inflammation. Blood.

[154] Stratmann A., Risau W., Plate K.H. (1998). Cell type-specific expression of angiopoietin-1 and angiopoietin-2 suggests a role in glioblastoma angiogenesis. Am. J. Pathol..

[155] Collazos-Aleman J.D., Gnecco-Gonzalez S., Jaramillo-Zarama B., Jimenez-Mora M.A., Mendivil C.O. (2022). The Role of Angiopoietins in Neovascular Diabetes-Related Retinal Diseases. Diabetes Ther..

[156] Zhang W., Yan H. (2012). Simvastatin increases circulating endothelial progenitor cells and reduces the formation and progression of diabetic retinopathy in rats. Exp. Eye Res..

[157] Lee S.G., Kim J.L., Lee H.K., Ryu G.W., Hur D.Y., Yun I.H., Yang J.W., Kim H.W. (2010). Simvastatin suppresses expression of angiogenic factors in the retinas of rats with streptozotocin-induced diabetes. Graefe’s Arch. Clin. Exp. Ophthalmol..

[158] Tuuminen R., Sahanne S., Loukovaara S. (2014). Low intravitreal angiopoietin-2 and VEGF levels in vitrectomized diabetic patients with simvastatin treatment. Acta Ophthalmol..

[159] Smith L.E., Wesolowski E., McLellan A., Kostyk S.K., D’Amato R., Sullivan R., D’Amore P.A. (1994). Oxygen-induced retinopathy in the mouse. Investg. Ophthalmol. Vis. Sci..

[160] Hangai M., Moon Y.S., Kitaya N., Chan C.K., Wu D.Y., Peters K.G., Ryan S.J., Hinton D.R. (2001). Systemically expressed soluble Tie2 inhibits intraocular neovascularization. Hum. Gene Ther..

[161] Jiang C., Ruan L., Zhang J., Huang X. (2018). Inhibitory Effects On Retinal Neovascularization by Ranibizumab and sTie2-Fc in An Oxygen-Induced Retinopathy Mouse Model. Curr. Eye Res..

[162] Li W., Zhang W., Zhang C., Zhu C., Yi X., Zhou Y., Lv Y. (2019). Soluble Tei2 fusion protein inhibits retinopathy of prematurity occurrence via regulation of the Ang/Tie2 pathway. Exp. Ther. Med..

[163] Lim H.S., Lip G.Y., Blann A.D. (2005). Angiopoietin-1 and angiopoietin-2 in diabetes mellitus: Relationship to VEGF, glycaemic control, endothelial damage/dysfunction and atherosclerosis. Atherosclerosis.

[164] Anuradha S., Mohan V., Gokulakrishnan K., Dixit M. (2010). Angiopoietin-2 levels in glucose intolerance, hypertension, and metabolic syndrome in Asian Indians (Chennai Urban Rural Epidemiology Study-74). Metabolism.

[165] Lip P.L., Chatterjee S., Caine G.J., Hope-Ross M., Gibson J., Blann A.D., Lip G.Y. (2004). Plasma vascular endothelial growth factor, angiopoietin-2, and soluble angiopoietin receptor tie-2 in diabetic retinopathy: Effects of laser photocoagulation and angiotensin receptor blockade. Br. J. Ophthalmol..

[166] You Q.Y., Zhuge F.Y., Zhu Q.Q., Si X.W. (2014). Effects of laser photocoagulation on serum angiopoietin-1, angiopoietin-2, angiopoietin-1/angiopoietin-2 ratio, and soluble angiopoietin receptor Tie-2 levels in type 2 diabetic patients with proliferative diabetic retinopathy. Int. J. Ophthalmol..

[167] Dieter C., Lemos N.E., de Faria Correa N.R., Costa A.R., Canani L.H., Crispim D., Bauer A.C. (2021). The rs2442598 polymorphism in the ANGPT-2 gene is associated with risk for diabetic retinopathy in patients with type 1 diabetes mellitus in a Brazilian population. Arq. Bras. de Endocrinol. Metabol..

[168] Regula J.T., Lundh von Leithner P., Foxton R., Barathi V.A., Gemmy Cheung C.M., Bo Tun S.B., Wey Y.S., Iwata D., Dostalek M., Moelleken J. (2017). Targeting key angiogenic pathways with a bispecific CrossMAb optimized for neovascular eye diseases. EMBO Mol. Med..

[169] Tuuminen R., Haukka J., Loukovaara S. (2015). Poor glycemic control associates with high intravitreal angiopoietin-2 levels in patients with diabetic retinopathy. Acta Ophthalmol..

[170] Klaassen I., Avery P., Schlingemann R.O., Steel D.H.W. (2022). Vitreous protein networks around ANG2 and VEGF in proliferative diabetic retinopathy and the differential effects of aflibercept versus bevacizumab pre-treatment. Sci. Rep..

[171] Mason R.H., Minaker S.A., Lahaie Luna G., Bapat P., Farahvash A., Garg A., Bhambra N., Muni R.H. (2022). Changes in aqueous and vitreous inflammatory cytokine levels in proliferative diabetic retinopathy: A systematic review and meta-analysis. Eye.

[172] Watanabe T. (1988). Raff MC: Retinal astrocytes are immigrants from the optic nerve. Nature.

[173] Tsai T., Alwees M., Asaad M.A., Theile J., Kakkassery V., Dick H.B., Schultz T., Joachim S.C. (2023). Increased Angiopoietin-1 and -2 levels in human vitreous are associated with proliferative diabetic retinopathy. PLoS ONE.

[174] Takagi H., Koyama S., Seike H., Oh H., Otani A., Matsumura M., Honda Y. (2003). Potential role of the angiopoietin/tie2 system in ischemia-induced retinal neovascularization. Investig. Ophthalmol. Vis. Sci..

[175] Surowka M., Schaefer W., Klein C. (2021). Ten years in the making: Application of CrossMab technology for the development of therapeutic bispecific antibodies and antibody fusion proteins. MAbs.

[176] Schaefer W., Regula J.T., Bahner M., Schanzer J., Croasdale R., Durr H., Gassner C., Georges G., Kettenberger H., Imhof-Jung S. (2011). Immunoglobulin domain crossover as a generic approach for the production of bispecific IgG antibodies. Proc. Natl. Acad. Sci. USA.

[177] Koenig P., Sanowar S., Lee C.V., Fuh G. (2017). Tuning the specificity of a Two-in-One Fab against three angiogenic antigens by fully utilizing the information of deep mutational scanning. MAbs.

[178] Sahni J., Patel S.S., Dugel P.U., Khanani A.M., Jhaveri C.D., Wykoff C.C., Hershberger V.S., Pauly-Evers M., Sadikhov S., Szczesny P. (2019). Simultaneous Inhibition of Angiopoietin-2 and Vascular Endothelial Growth Factor-A with Faricimab in Diabetic Macular Edema: BOULEVARD Phase 2 Randomized Trial. Ophthalmology.

[179] Eter N., Singh R.P., Abreu F., Asik K., Basu K., Baumal C., Chang A., Csaky K.G., Haskova Z., Lin H. (2022). YOSEMITE and RHINE: Phase 3 Randomized Clinical Trials of Faricimab for Diabetic Macular Edema: Study Design and Rationale. Ophthalmol. Sci..

[180] Wykoff C.C., Abreu F., Adamis A.P., Basu K., Eichenbaum D.A., Haskova Z., Lin H., Loewenstein A., Mohan S., Pearce I.A. (2022). Efficacy, durability, and safety of intravitreal faricimab with extended dosing up to every 16 weeks in patients with diabetic macular oedema (YOSEMITE and RHINE): Two randomised, double-masked, phase 3 trials. Lancet.

[181] Ishida S., Chen S.J., Murata T., Ogura Y., Ruamviboonsuk P., Sakamoto T., Fujita T., Kawano M., Ohsawa S., Abreu F. (2023). Efficacy, Durability, and Safety of Faricimab in Patients From Asian Countries With Diabetic Macular Edema: 1-Year Subgroup Analysis of the Phase III YOSEMITE and RHINE Trials. Asia-Pac. J. Ophthalmol..

[182] Wong T.Y., Haskova Z., Asik K., Baumal C.R., Csaky K.G., Eter N., Ives J.A., Jaffe G.J., Korobelnik J.F., Lin H. (2023). Faricimab Treat-and-Extend for Diabetic Macular Edema: 2-Year Results from the Randomized Phase 3 YOSEMITE and RHINE Trials. Ophthalmology.

[183] Watkins C., Paulo T., Buhrer C., Holekamp N.M., Bagijn M. (2023). Comparative Efficacy, Durability and Safety of Faricimab in the Treatment of Diabetic Macular Edema: A Systematic Literature Review and Network Meta-Analysis. Adv. Ther..

[184] Li G., Zhu N., Ji A. (2023). Comparative efficacy and safety of Faricimab and other anti-VEGF therapy for age-related macular degeneration and diabetic macular edema: A systematic review and meta-analysis of randomized clinical trials. Medicine.

[185] Rush R.B. (2023). One Year Results of Faricimab for Aflibercept-Resistant Diabetic Macular Edema. Clin. Ophthalmol..

[186] Rush R.B., Rush S.W. (2022). Faricimab for Treatment-Resistant Diabetic Macular Edema. Clin. Ophthalmol..

[187] Ohara H., Harada Y., Hiyama T., Sadahide A., Minamoto A., Kiuchi Y. (2023). Faricimab for Diabetic Macular Edema in Patients Refractory to Ranibizumab or Aflibercept. Medicina.

[188] Takamura Y., Yamada Y., Morioka M., Gozawa M., Matsumura T., Inatani M. (2023). Turnover of Microaneurysms After Intravitreal Injections of Faricimab for Diabetic Macular Edema. Investig. Ophthalmol. Vis. Sci..

[189] Kusuhara S., Kishimoto-Kishi M., Matsumiya W., Miki A., Imai H., Nakamura M. (2023). Short-Term Outcomes of Intravitreal Faricimab Injection for Diabetic Macular Edema. Medicina.

[190] Sawa M., Nakagawa N., Shunto T., Nishiyama I. (2024). Two cases of diabetic macular edema with diminished areas of retinal non-perfusion and microaneurysms after intravitreal faricimab injections. Am. J. Ophthalmol. Case Rep..

[191] Brown D.M., Boyer D.S., Csaky K., Vitti R., Perlee L., Chu K.W., Asmus F., Leal S., Zeitz O., Cheng Y. (2022). Intravitreal Nesvacumab (Antiangiopoietin 2) Plus Aflibercept in Diabetic Macular Edema: Phase 2 RUBY Randomized Trial. Retina.

[192] Heier J.S., Ho A.C., Boyer D.S., Csaky K., Vitti R., Perlee L., Chu K.W., Asmus F., Leal S., Zeitz O. (2022). Intravitreal Nesvacumab (Anti-Angiopoietin-2) Plus Aflibercept in Neovascular AMD: Phase 2 ONYX Randomized Trial. J. Vitr. Dis..

[193] Campochiaro P.A., Sophie R., Tolentino M., Miller D.M., Browning D., Boyer D.S., Heier J.S., Gambino L., Withers B., Brigell M. (2015). Treatment of diabetic macular edema with an inhibitor of vascular endothelial-protein tyrosine phosphatase that activates Tie2. Ophthalmology.

[194] Campochiaro P.A., Peters K.G. (2016). Targeting Tie2 for Treatment of Diabetic Retinopathy and Diabetic Macular Edema. Curr. Diab. Rep..

[195] Campochiaro P.A., Khanani A., Singer M., Patel S., Boyer D., Dugel P., Kherani S., Withers B., Gambino L., Peters K. (2016). Enhanced Benefit in Diabetic Macular Edema from AKB-9778 Tie2 Activation Combined with Vascular Endothelial Growth Factor Suppression. Ophthalmology.

[196] Akwii R.G., Mikelis C.M. (2021). Targeting the Angiopoietin/Tie Pathway: Prospects for Treatment of Retinal and Respiratory Disorders. Drugs.

[197] Van Hove I., Hu T.T., Beets K., Van Bergen T., Etienne I., Stitt A.W., Vermassen E., Feyen J.H.M. (2021). Targeting RGD-binding integrins as an integrative therapy for diabetic retinopathy and neovascular age-related macular degeneration. Prog. Retin. Eye Res..

[198] Parsons J.T., Horwitz A.R., Schwartz M.A. (2010). Cell adhesion: Integrating cytoskeletal dynamics and cellular tension. Nat. Rev. Mol. Cell Biol..

[199] Cascone I., Audero E., Giraudo E., Napione L., Maniero F., Philips M.R., Collard J.G., Serini G., Bussolino F. (2003). Tie-2-dependent activation of RhoA and Rac1 participates in endothelial cell motility triggered by angiopoietin-1. Blood.

[200] Jones N., Iljin K., Dumont D.J., Alitalo K. (2001). Tie receptors: New modulators of angiogenic and lymphangiogenic responses. Nat. Rev. Mol. Cell Biol..

[201] Kontos C.D., Stauffer T.P., Yang W.P., York J.D., Huang L., Blanar M.A., Meyer T., Peters K.G. (1998). Tyrosine 1101 of Tie2 is the major site of association of p85 and is required for activation of phosphatidylinositol 3-kinase and Akt. Mol. Cell. Biol..

[202] Silva R.L.E., Kanan Y., Mirando A.C., Kim J., Shmueli R.B., Lorenc V.E., Fortmann S.D., Sciamanna J., Pandey N.B., Green J.J. (2017). Tyrosine kinase blocking collagen IV-derived peptide suppresses ocular neovascularization and vascular leakage. Sci. Transl. Med..

[203] Mirando A.C., Shen J., Silva R.L.E., Chu Z., Sass N.C., Lorenc V.E., Green J.J., Campochiaro P.A., Popel A.S., Pandey N.B. (2019). A collagen IV-derived peptide disrupts alpha5beta1 integrin and potentiates Ang2/Tie2 signaling. JCI Insight.

[204] Nguyen Q.D., Heier J.S., Do D.V., Mirando A.C., Pandey N.B., Sheng H., Heah T. (2020). The Tie2 signaling pathway in retinal vascular diseases: A novel therapeutic target in the eye. Int. J. Retin. Vitr..

[205] Xu L., Estelles A., Briante R., Kurtzman A.L., Hannum C.H., Kashyap A.K., Horowitz L., Horowitz M., Bhatt R.R., Lerner R.A. (2010). Surrobodies with functional tails. J. Mol. Biol..

[206] Xu L., Yee H., Chan C., Kashyap A.K., Horowitz L., Horowitz M., Bhatt R.R., Lerner R.A. (2008). Combinatorial surrobody libraries. Proc. Natl. Acad. Sci. USA.

